# Heterochrony and Early Left-Right Asymmetry in the Development of the Cardiorespiratory System of Snakes

**DOI:** 10.1371/journal.pone.0116416

**Published:** 2015-01-02

**Authors:** Benjamin J. van Soldt, Brian D. Metscher, Robert E. Poelmann, Bart Vervust, Freek J. Vonk, Gerd B. Müller, Michael K. Richardson

**Affiliations:** 1 Institute of Biology, University of Leiden, Leiden, the Netherlands; 2 Department of Theoretical Biology, University of Vienna, Vienna, Austria; 3 Department of Anatomy and Embryology, Leiden University Medical Center, Leiden, the Netherlands; 4 Department of Biology, University of Antwerp, Antwerp, Belgium; 5 NCB Naturalis, Leiden, the Netherlands; Zhejiang University, China

## Abstract

Snake lungs show a remarkable diversity of organ asymmetries. The right lung is always fully developed, while the left lung is either absent, vestigial, or well-developed (but smaller than the right). A ‘tracheal lung’ is present in some taxa. These asymmetries are reflected in the pulmonary arteries. Lung asymmetry is known to appear at early stages of development in *Thamnophis radix* and *Natrix natrix*. Unfortunately, there is no developmental data on snakes with a well-developed or absent left lung. We examine the adult and developmental morphology of the lung and pulmonary arteries in the snakes *Python curtus breitensteini*, *Pantherophis guttata guttata*, *Elaphe obsoleta spiloides*, *Calloselasma rhodostoma* and *Causus rhombeatus* using gross dissection, MicroCT scanning and 3D reconstruction. We find that the right and tracheal lung develop similarly in these species. By contrast, the left lung either: (1) fails to develop; (2) elongates more slowly and aborts early without (2a) or with (2b) subsequent development of faveoli; (3) or develops normally. A right pulmonary artery always develops, but the left develops only if the left lung develops. No pulmonary artery develops in relation to the tracheal lung. We conclude that heterochrony in lung bud development contributes to lung asymmetry in several snake taxa. Secondly, the development of the pulmonary arteries is asymmetric at early stages, possibly because the splanchnic plexus fails to develop when the left lung is reduced. Finally, some changes in the topography of the pulmonary arteries are consequent on ontogenetic displacement of the heart down the body. Our findings show that the left-right asymmetry in the cardiorespiratory system of snakes is expressed early in development and may become phenotypically expressed through heterochronic shifts in growth, and changes in axial relations of organs and vessels. We propose a step-wise model for reduction of the left lung during snake evolution.

## Introduction

Pulmonary left-right asymmetry is an intriguing deviation from what is otherwise a mostly bilaterally symmetric body plan in vertebrates. To date, studies have focused on the developmental mechanisms of pulmonary asymmetry in mouse [1]–[3]. These studies show critical roles for Pitx2 [4], Tbx4 and Tbx5 [5]. In addition, studies in *Drosophila*, mouse and birds suggest that mechanisms of pulmonary development are highly conserved throughout the animal kingdom [6], [7]. For example, *Drosophila branchless* and *breathless* are homologues to mammalian Fgf10 and Fgfr2b, respectively [8], [9]. It thus seems likely that developmental mechanisms governing pulmonary left-right asymmetry in other species are similar to those in the mouse, so that current data may be used to study developmental mechanisms in other species.

Developmental mechanisms of pulmonary left-right asymmetry are not well understood in species groups outside mammals, although this is where many striking instances of this anatomical feature are found. One example is the snake respiratory system (see for reviews Refs. [10]–[14]). Briefly, the right lung in snakes is typically well-developed, while the left lung exhibits a range of anatomies that can be subdivided into an absent (type one), a vestigial (type two) and a well-developed type (type three) [12] (Table 1). The pattern of the pulmonary arteries varies in ways similar to the lungs (Table 1).

**Table 1:** Overview of some variations in the morphology of the lungs in selected snake taxa based on the literature.

	*Left lung*					*Right lung*			*Tracheal lung*	
Character	Adult anatomy			Length of vascular portion of left lung	Length of saccular portion of left lung	Adult anatomy	Length of vascular portion of left lung	Length of saccular portion of left lung	Adult anatomy	
States [10], [12], [14], [75]	Absent	Vestigial	Well-developed	Varies widely depending on species	Varies widely depending on species	Well-developed	Varies widely depending on species	Varies widely depending on species	Present	Absent
State of associated pulmonary artery [54]–[56], [60], [71], [76]–[82]	Absent left pulmonary artery	Absent or reduced left pulmonary artery	Present left pulmonary artery	Depending on species, left pulmonary artery extends throughout the entire vascular lung, or is overtaken by right pulmonary artery.	No left pulmonary artery associated with saccular left lung	Present right pulmonary artery	Right pulmonary artery extends throughout all of the vascular lung	No right pulmonary artery associated with saccular right lung	Present anterior pulmonary artery	Absent anterior pulmonary artery
Representative taxa	Acrochordidae	Colubridae	Boidae			All examined snakes			Acrochordidae	Boidae

The various types of snake lung anatomies suggest distinct genetic alterations of the ancestral mechanism of pulmonary development. By investigating the development of these lung anatomies and comparing them to relevant outgroups, we may get a better understanding of the evolution of the snake respiratory system. A key question herein is which snake lung anatomy type represents the primitive condition. Previous work has designated the well-developed left lung (type three) as such [10]. Another study presents the vestigial left lung as being the primitive condition [12]. Currently there is no consensus on the verity of either of these hypotheses.

**Figure 1:**
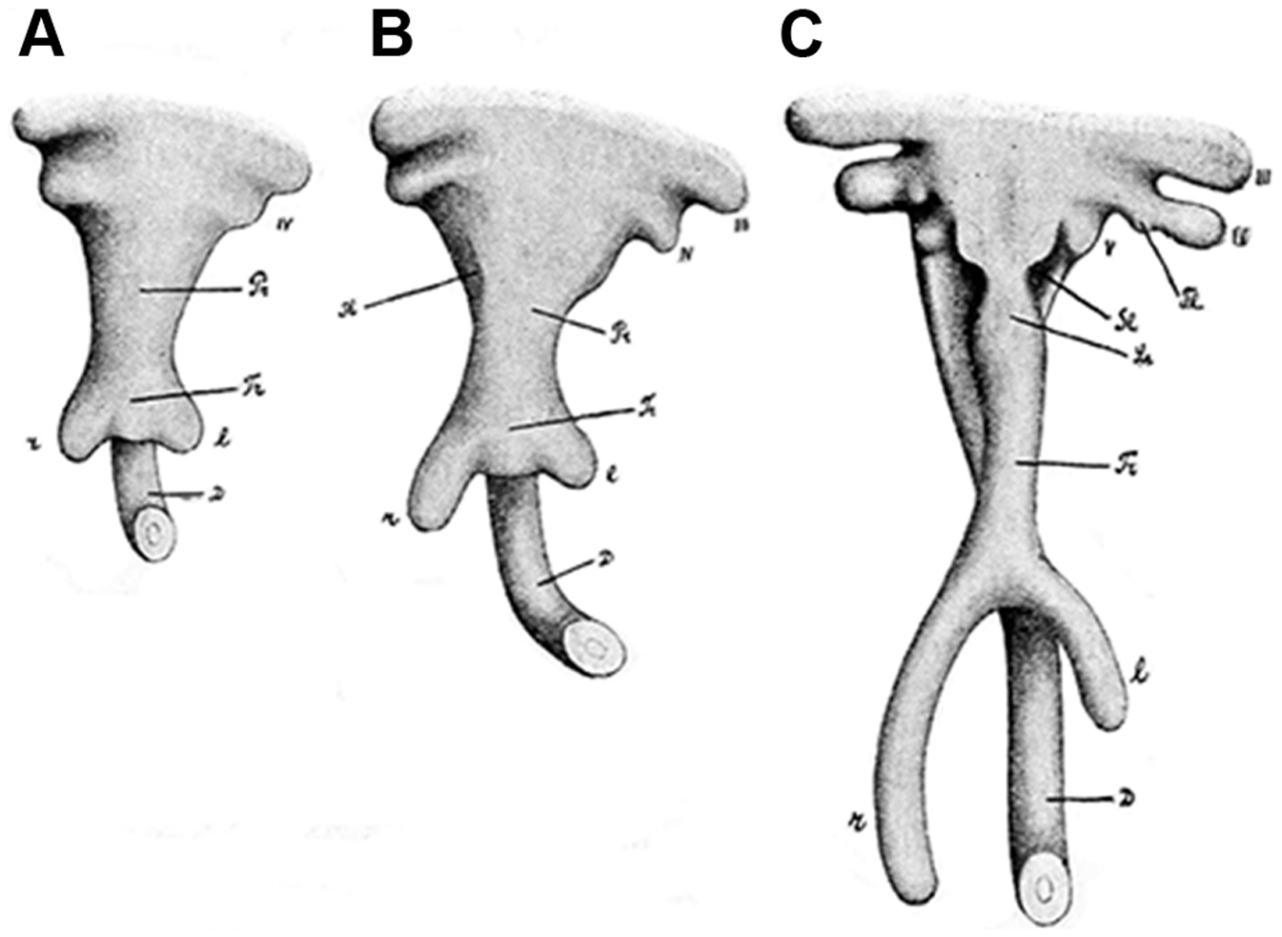
Early lung development in *Natrix natrix* according to Schmalhausen. Overview figure showing three subsequent stages of lung development in the snake *Natrix natrix*, a snake reported [12] to have a vestigial left lung. d: esophagus; l  =  left lung bud; r  =  right lung bud; tr  =  trachea. Adapted from Figures 5–7 in Ref. [15].

Schmalhausen [15] found that in the European grass snake *Natrix natrix* Linnaeus, 1758 (family Colubridae) (well-developed right lung and vestigial left lung [12]) the early lung bud is an evagination from the floor of the endodermal foregut, which then bifurcates into two bronchial buds (Fig. 1A). This early pattern is similar to that described in the mouse and human [3], [16], [17]). However, Schmalhausen stated that the right bronchial bud elongates more rapidly than the left one does (Fig. 1B, C) and continues growing at a later developmental stage than the left, whose growth ends prematurely. This ‘truncation’ of development is one kind of heterochrony (change in developmental sequence during evolution; reviewed in Refs. [18]–[23]). Similar findings were found in the Plains garter snake *Thamnophis radix* Baird and Girard, 1853 (family Colubridae) [24], which has an adult pulmonary anatomy similar to that of *Natrix natrix* (see also Ref. [12] for a review). While these studies suggest that pulmonary asymmetry is determined during early stages of lung development by heterochrony, without developmental data on snake species with a well-developed or absent left lung in the adult, the issue remains unresolved.

Regarding pulmonary artery development, studies in chick, mouse and man [2], [25]–[31] show that the pulmonary arteries develop from splanchnic plexi, surrounding the developing lung buds [25], [30]–[32]. When the pulmonary trunk and the sixth pharyngeal arch artery (PAA) have formed, microvasculature from the lungs connects with this PAA pair, the two coming into vascular continuity. Stabilization by smooth muscle cells forms the definitive pulmonary artery [25], [31], [33]. With exception of establishment of the pulmonary trunk [34]–[39], little is known about these events in reptiles other than birds.

In this study, we first categorize the pulmonary artery and lung anatomies of adult snakes from a range of taxa. We then use these typifications to examine the development of the snake lung and pulmonary artery, using series of embryos of five species of snake: (i) Malayan pit viper *Calloselasma rhodostoma* Kuhl, 1824, family Viperidae; (ii) common night adder *Causus rhombeatus* Liechtenstein, 1823, family Viperidae; (iii) corn snake *Pantherophis guttata guttata* Linnaeus, 1766, family Colubridae; (vi) midland rat snake *Elaphe obsoleta spiloides* Duméril, 1854, family Colubridae; and (v) blood python *Python curtus breitensteini* Schlegel, 1872, family Pythonidae. Due to limited material availability, we use a 22 days-after-oviposition (dao) specimen of *Elaphe obsoleta spiloides* to complement the *Pantherophis guttata guttata* specimens, since these snake species have similar pulmonary anatomies [12]. The same applies to *Calloselasma rhodostoma* and *Causus rhombeatus*. However, *Causus rhombeatus* is known to have a thoraconuchal lung (continuous tracheal/right lung), while in *Calloselasma rhodostoma* tracheal and right lung are distinct [12].

## Materials and Methods

### Ethics Statement

Under the active Dutch legislation at the time the study was performed (2011–2012), experiments with snake embryos were not considered to be animal experiments while the embryo was not free feeding, i.e. pre-hatching (Art. 1, Experiments on Animals Act (Wet op de Dierproeven)). Ethical review and advice of the animal experiments committee was therefore not required for this study. Still, the Leiden University animal welfare officer was approached for advice and approval. Dutch animal experimentation legislation is based on the Guidelines on the protection of experimental animals by the Council of Europe, Directive 86/609/EEC.

Adult (Table 2) and embryonic (Table 3) snakes were from the zoology collection of the Institute of Biology, University of Leiden. Adult snakes were donated to the Institute of Biology by Dutch or Belgian pet owners as carcasses after the animals had died from natural causes. Specimens were fixed and stored in 70% ethanol upon donation. Embryos of *Causus rhombeatus and Calloselasma rhodostoma* were donated to the Institute of Biology; the other species' embryos were purchased from Dutch pet owners. All embryos were acquired while still alive, incubated at 28°C in a Heraeus B5060E incubator (Hanau, Germany) and euthanized instantly at various stages by fixating them in 4% paraformaldehyde (PFA) overnight at 4°C. They were then dehydrated in methanol, staged according to Zehr [40] (cross-checked with other staging tables [41]–[45]) and stored in 100% methanol at −20°C. For ages of euthanization, see Table 3. Incubation times vary wildly between snake species and are largely dependent on incubation temperatures. In our hands, at temperatures between 25°C and 29°C: *Calloselasma rhodostoma* embryos hatch after 32 – 39 days, *Causus rhombeatus* after 75 – 80 days, *Elaphe obsoleta spiloides* after 50 – 76 days, *Pantherophis gutta guttata* after 53 – 62 days, and *Python curtus breitensteini* after 58 – 65 days (at 32°C).

**Table 2:** Snake specimens used here to study snake lungs and pulmonary artery anatomy and the associated results.

Species	Number analyzed	Common name	Family	Subfamily	Tracheal lung	Right lung anatomy	Left lung anatomy	PA* type
*Acrochordus granulatus*	2	Wart snake	Acrochordidae	N/A	Present	Full	Absent	PA1
*Eunectes notaeus*	1	Yellow anaconda	Boidae	Boinae	Absent	Full	Full	PA3
*Morelia viridis*	2	Green tree python	Boidae	Pythoninae	Absent	Full	Full	PA3
*Python regius*	5	Ball python	Boidae	Pythoninae	Absent	Full	Full	PA3
*Lampropeltis triangulum sinaloae*	1	Scarlet kingsnake	Colubridae	Colubrinae	?	Full	Vestigial	PA2
*Pantherophis guttata guttata*	7	Corn snake	Colubridae	Colubrinae	Present	Full	Vestigial	PA2
*Bungarus candidus*	1	Blue krait	Elapidae	Elapinae	Present	Full	Vestigial	PA2
*Bungarus fasciatus*	1	Banded krait	Elapidae	Elapinae	Yes	Full	Vestigial	PA2
*Hydrophis elegans*	3	Elegant sea-snake	Elapidae	Hydrophiinae	Yes	Full	Absent	PA1
*Natrix tessellata*	3	Dice snake	Natricidae	Natricinae	Yes	Full	Vestigial	PA2
*Thamnophis sirtalis concinnus*	1	Common garter snake	Natricidae	Natricinae	Absent	Full	Vestigial	PA2
*Calloselasma rhodostoma*	3	Malayan pit viper	Viperidae	Crotalinae	Yes	Full	Vestigial	PA1
*Trimeresurus sp.*	1	Pit viper	Viperidae	Crotalinae	Yes	Full	Vestigial	PA1

*PA  =  Pulmonary artery.

**Table 3:** Snake embryo specimens used here to study the development of the snake pulmonary arteries and lungs.

Species	Age (dao*)	Zehr stage [40]	Jackson stage [41]	Khannoon stage [42]	Boughner/Buchtová stage [44], [45]	Lung anatomy type [12]	PA** type
*Calloselasma rhodostoma*	3.00	26	1	2	1	Type 1	PA1
*Calloselasma rhodostoma*	20.00	36/37	8	8	7/8	Type 1	PA1
*Causus rhombeatus*	5.00	25	1	1	1	Type 1	PA1
*Causus rhombeatus*	8.00	28	1/2	2	1/3	Type 1	PA1
*Causus rhombeatus*	10.00	30	3	3/4	3	Type 1	PA1
*Elaphe obsoleta spiloides*	22.00	34	8	6	6/7	Type 2	PA2
*Pantherophis guttata guttata*	2.00	25	1	1	1	Type 2	PA2
*Pantherophis guttata guttata*	6.00	26	2	2	1/3	Type 2	PA2
*Pantherophis guttata guttata*	9.00	27	3	3	3	Type 2	PA2
*Pantherophis guttata guttata*	12.00	33	5	5/6	4/6	Type 2	PA2
*Python curtus breitensteini*	2.00	24	1	1	1	Type 3	PA3
*Python curtus breitensteini*	5.00	28	2	2	1/3	Type 3	PA3
*Python curtus breitensteini*	10.00	30	3/4	3	3/4	Type 3	PA3
*Python curtus breitensteini*	14.00	32	5	4	4	Type 3	PA3

*dao  =  days after oviposition.

**PA  =  Pulmonary Artery.

### Pulmonary artery typifications

**Figure 2:**
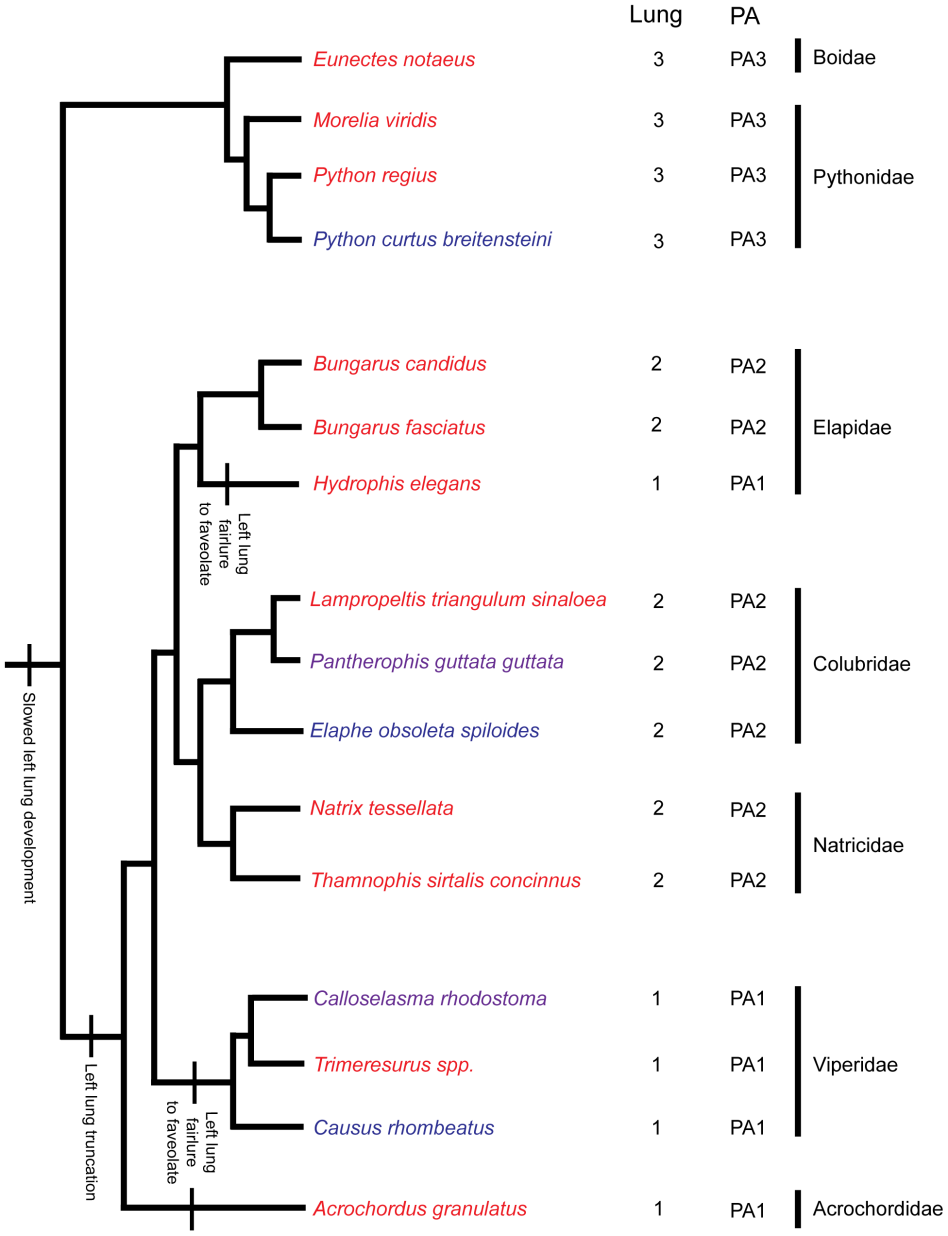
Phylogeny of species used, with our hypothesis of the lung reduction steps mapped on. Red species were used for lung typification only, blue species for lung and vascular development only, and purple species for both. ‘Lung’ and ‘PA’ give lung type [12] and pulmonary artery type (Fig. 4), respectively. Stem and family branches are based on Refs. [46]–[48], [50]; the branches within families are based on Refs. [12], [49], [50].

Adult snakes were measured (snout to cloaca and cloaca to tail-tip) and subsequently opened using a ventral midline incision using standard dissection equipment. The mesenteries were removed to expose the viscera. The heart, lung or lungs, and great vessels were examined by gross dissection, both *in situ* and after removal from the animal. A phylogeny of all the species studied, based on Refs. [12], [46]–[50], is shown in Fig. 2. For lung and pulmonary artery typifications in adult snakes we gathered data from 28 published studies (S1 Table) and our own dissections.

### MicroCT imaging and Amira segmentation

**Figure 3:**
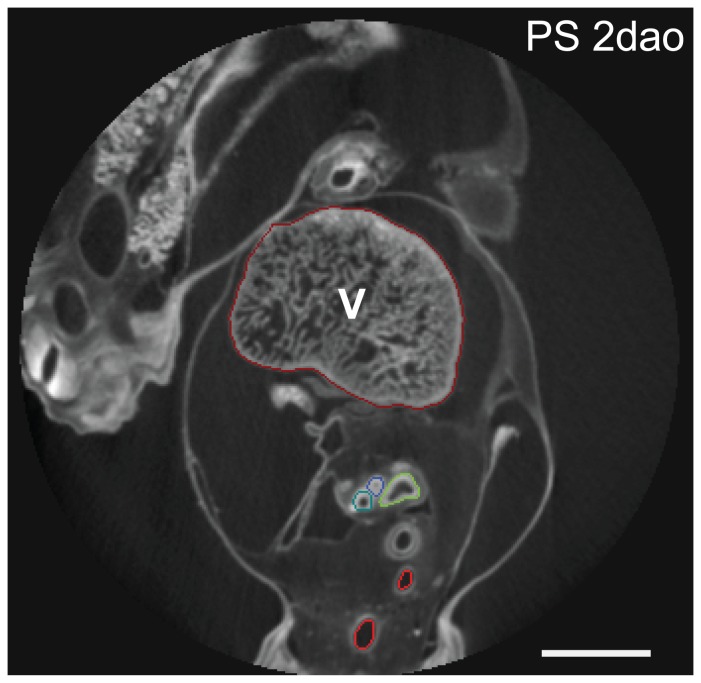
Example of annotation methods in Amira 5.4.5. Dorsal side is up. V  =  ventricle. Scale bar represents 1 mm.

For x-ray contrast enhancement, embryos were stained for one to four days in 1% phosphotungstic acid (PTA) dissolved in 100% methanol [51], [52]. Samples were then brought back to clean 100% methanol and mounted for scanning in plastic tubes or large pipette tips [53]. Attenuation-based microtomographic images were made using an Xradia MicroXCT system (Carl Zeiss X-Ray Microscopy, Pleasanton, CA), with a tungsten source (Hamamatsu L9421-02) set at 40 kV and 100 mA (4W) for a nominal spot size of 5µm. Projection images were taken every 0.2° over a rotation of 180° plus the imaging cone angle (usually 2–6° – thus 921–961 images per scan). Images were acquired with pixel sizes of 4–10µm, and tomographic reconstructions were made with the resident software (XMReconstructor) using a beam-hardening correction of 0.4 and 2×2 binning, resulting in final voxel sizes of 8–20µm. Reconstructed images were exported as TIFF and loaded into Amira version 5.4 for annotation. We annotated the lumina of the blood vessels and lungs (Fig. 3; bright red outlines), but in the youngest stages we included epithelium (Fig. 3; astral blue, dark yellow and dark red outlines).

## Results

### Classification of the adult pulmonary arteries

Based on our dissections (Table 2) and the literature (S1 Table), all snakes have a single arterial pulmonary trunk arising from the ventricle. When it reaches roughly the top of the left atrium, the pulmonary artery may bifurcate, so that we can distinguish pulmonary artery anatomies based on two key characteristics of the vessels: (i) The number of pulmonary arteries: there may be one or two primary branches, and (ii) The direction in which the vessels run: they run either anteriorly or posteriorly.

**Figure 4:**
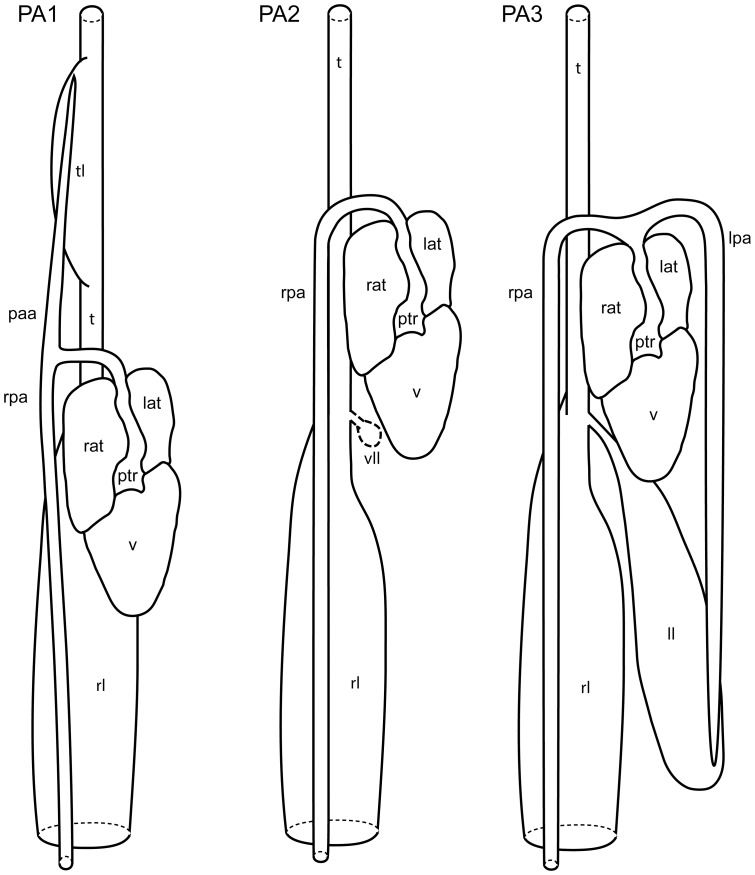
Schematic portrayal of pulmonary artery types and the lungs to which they connect. Ventral views. PA1: pulmonary trunk that bifurcates into an ascending branch (connects with tracheal lung; tl) and a descending branch on the right (connects with the right lung; rl). PA2: single pulmonary artery, descending posteriorly and connecting to the right lung (rl); PA3: pulmonary trunk that bifurcates into two descending branches, each connecting to its respective lung (rl and ll); lat  =  left atrium; lpa  =  left pulmonary artery; pa  =  pulmonary artery; paa  =  anterior pulmonary artery; rat  =  right atrium; ptr  =  pulmonary trunk; rpa  =  right pulmonary artery; t  =  trachea; v  =  ventricle.

**Figure 5:**
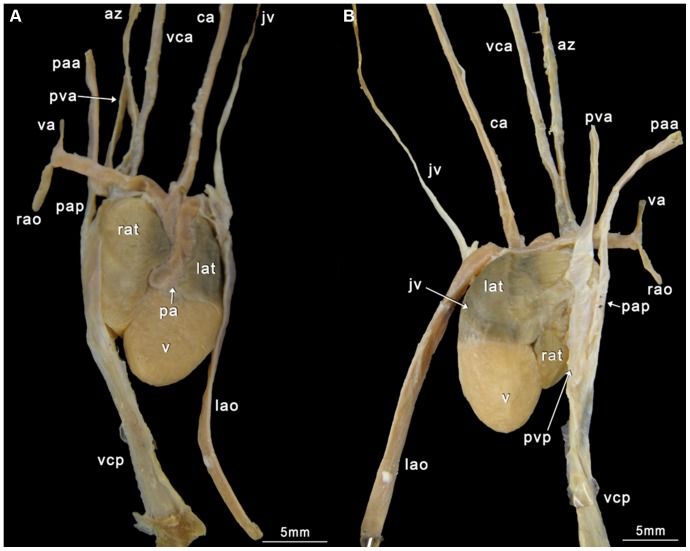
The *Trimeresurus* spp. Heart (ID PK07). A Ventral view; B Dorsal view. az  =  azygos vein; ca  =  carotid artery; jv  =  jugular vein; lao  =  left aorta; lat  =  left atrium; lu  =  lung; paa  =  pulmonary artery anterior; pap  =  pulmonary artery posterior; pva  =  pulmonary vein anterior; pvp  =  pulmonary vein posterior; rao  =  right aorta; rat  =  right atrium; v  =  ventricle; va  =  vertebral artery; vca  =  vena cava anterior; vcp  =  vena cava posterior.

**Figure 6:**
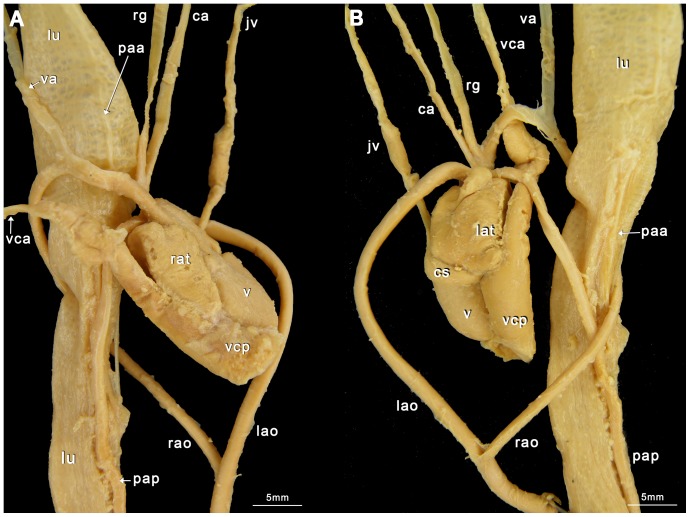
The *Hydrophis elegans* Heart (ID BS22). A Ventral view; B Dorsal view. ca  =  carotid artery; jv  =  jugular vein; lao  =  left aorta; lat  =  left atrium; lu  =  lung; paa  =  pulmonary artery anterior; pap  =  pulmonary artery posterior; pva  =  pulmonary vein anterior; pvp  =  pulmonary vein posterior; rao  =  right aorta; rat  =  right atrium; v  =  ventricle; va  =  vertebral artery; vca  =  vena cava anterior; vcp  =  vena cava posterior.

**Figure 7:**
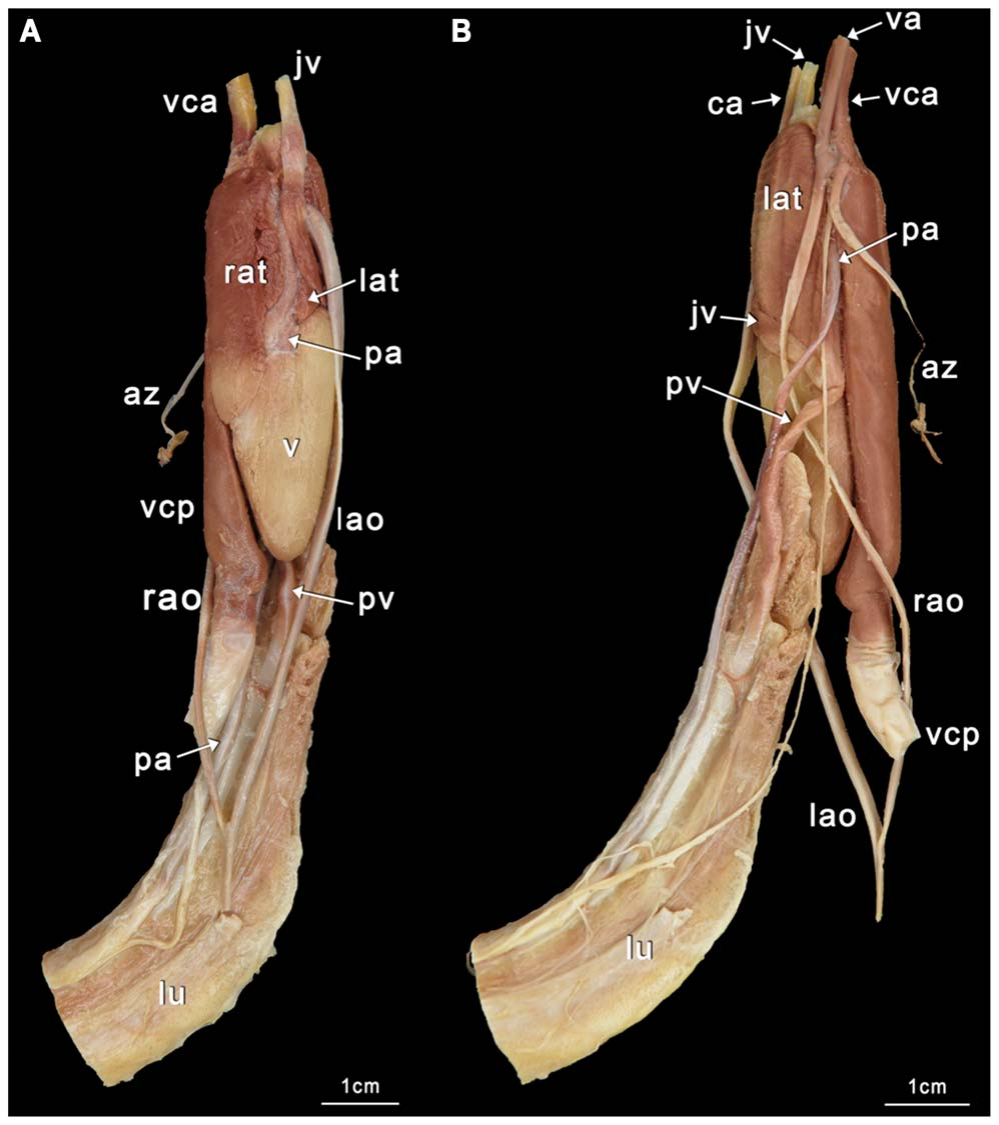
The *Bungarus candidus* heart (ID BS24). A Ventral view; B Dorsal view. The lung was turned laterally by 180 degrees for the purpose of these photographs, which has detached the pulmonary vein and vena cava posterior that normally run together. az  =  azygous artery; ca  =  carotid artery; jv  =  jugular vein; lao  =  left aorta; lat  =  left atrium; lu  =  lung; pa  =  pulmonary artery; pv  =  pulmonary vein; rao  =  right aorta; rat  =  right atrium; v  =  ventricle; va  =  vertebral artery; vca  =  vena cava anterior; vcp  =  vena cava posterior.

**Figure 8:**
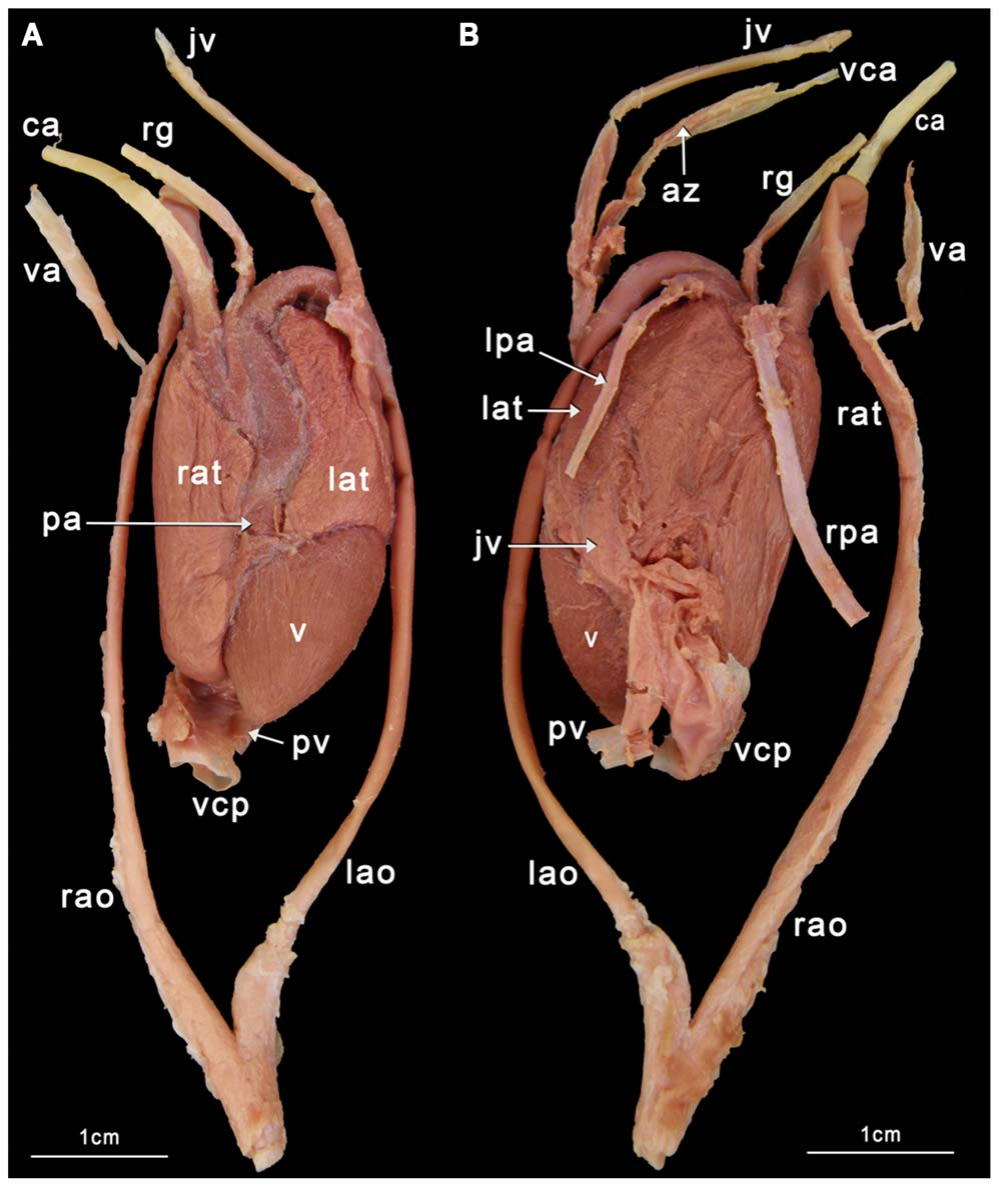
The *Eunectus notaeus* heart (ID BS8). A ventral view; B dorsal view. az  =  azygos vein; ca  =  carotid artery; jv  =  jugular vein; lao  =  left aorta; lat  =  left atrium; pv  =  pulmonary vein; lpa  =  left pulmonary artery; rao  =  right aorta; rat  =  right atrium; rpa  =  right pulmonary artery; rg  =  ramus glandularis; v  =  ventricle; va  =  vertebral artery; vca  =  vena cava anterior; vcp  =  vena cava posterior.

Our classification is summarized in Table 4. Schematic illustrations showing these types can be seen in Fig. 4, and photographs showing typical examples of the types can be seen in Figs. 5–8. As we aimed for a generalized, gross typification, we only took the main branches into account. See Fig. 2 for a phylogeny that summarizes the results for the snakes dissected by us.

**Table 4:** Classification of observed pulmonary artery anatomies.

Our classification	Pulmonary trunk	Taxonomic distribution in our samples	Figures
PA1	Trunk curves to the right, then bifurcates into anterior and posterior branches	Hydrophiinae, Acrochordidae and Crotalinae (*Acrochordus granulatus, Hydrophis elegans, Calloselasma rhodostoma, Trimeresurus sp.*)	5B, 6A,B
PA2	No bifurcation; continues as a single, descending pulmonary artery	Colubrinae, Natricinae and Elapinae (*Lampropeltis triangulum sinaloae, Pantherophis guttata guttata, Bungarus candidus, Bungarus fasciatus, Natrix tessellata*)	7A,B
PA3	Bifurcates into right and left branches descending on the dorsal side of the heart	Boinae and Pythoninae (*Eunectes notaeus, Morelia viridis, Python regius*)	8A,B

### Embryonic development of the lungs and pulmonary arteries

**Figure 9:**
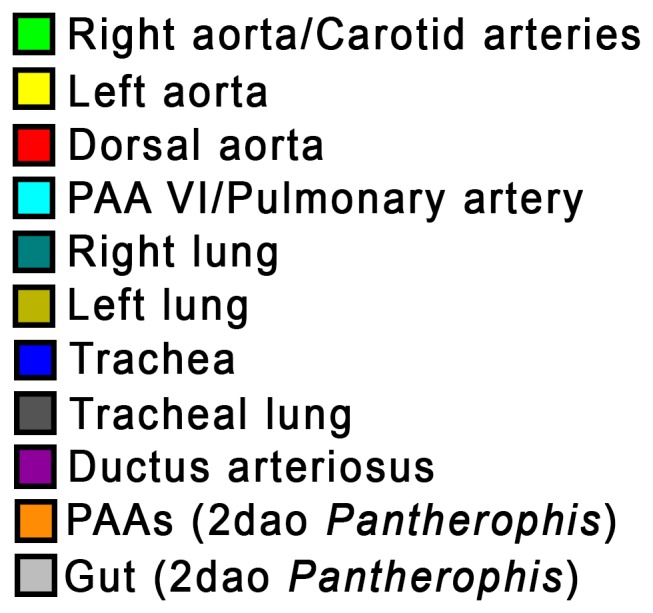
Legend for 3D segmentations in Amira.

**Figure 10:**
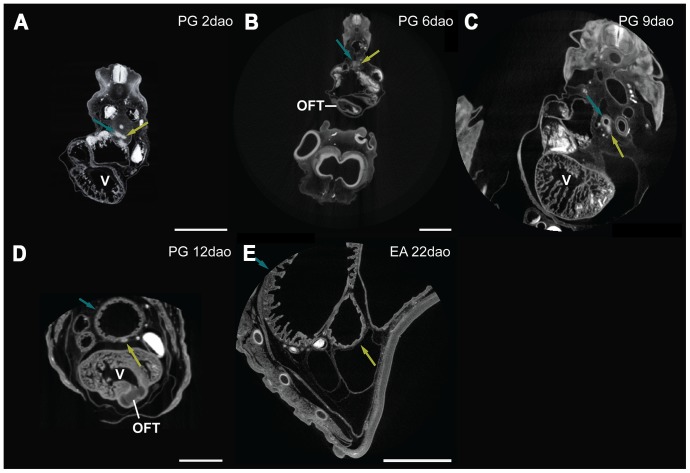
MicroCT transverse sections of embryos showing lung lumina. Sections are of *Pantherophis guttata guttata* (A – D) and *Elaphe obsoleta spiloides* (E). The embryo's dorsal sides are up. Astral blue arrows denote right lung, dark yellow left lung; V  =  ventricle; OFT  =  outflow tract. Annotation done in Amira 5.4.5. Scale bar represents 1 mm.

**Figure 11:**
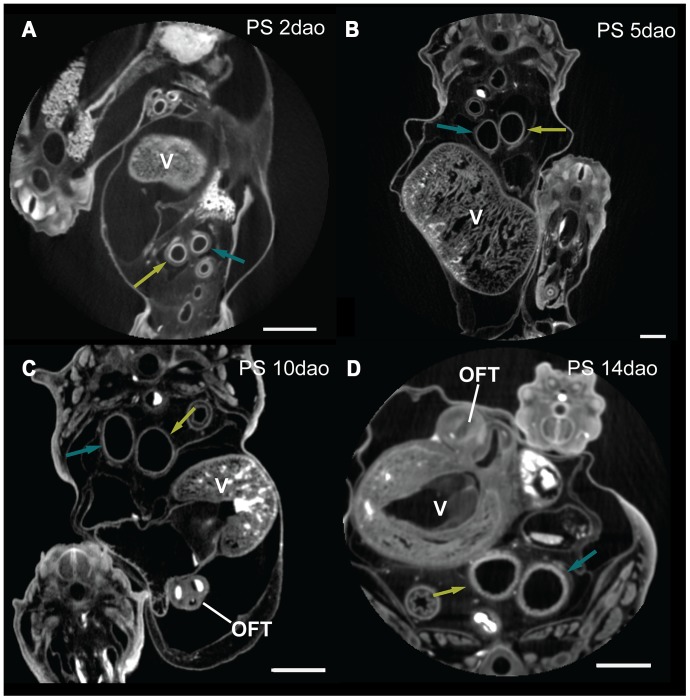
MicroCT transverse sections of embryos showing lung lumina. Sections are of *Python curtus breitensteini*. The embryo's dorsal sides are up. Astral blue arrows denote right lung, dark yellow left lung; V  =  ventricle; OFT  =  outflow tract. Annotation done in Amira 5.4.5. Scale bar represents 1 mm.

**Figure 12:**
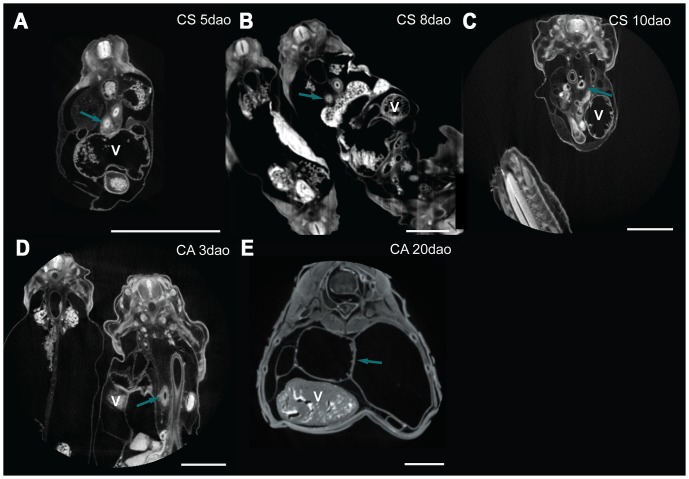
MicroCT transverse sections of embryos showing lung lumina. Sections are of *Causus rhombeatus* (A – C) and *Calloselasma rhodostoma* (D, E). The embryo's dorsal sides are up. Astral blue arrows denote right lung, dark yellow left lung; V  =  ventricle. Annotation done in Amira 5.4.5. Scale bar represents 1 mm.

**Figure 13:**
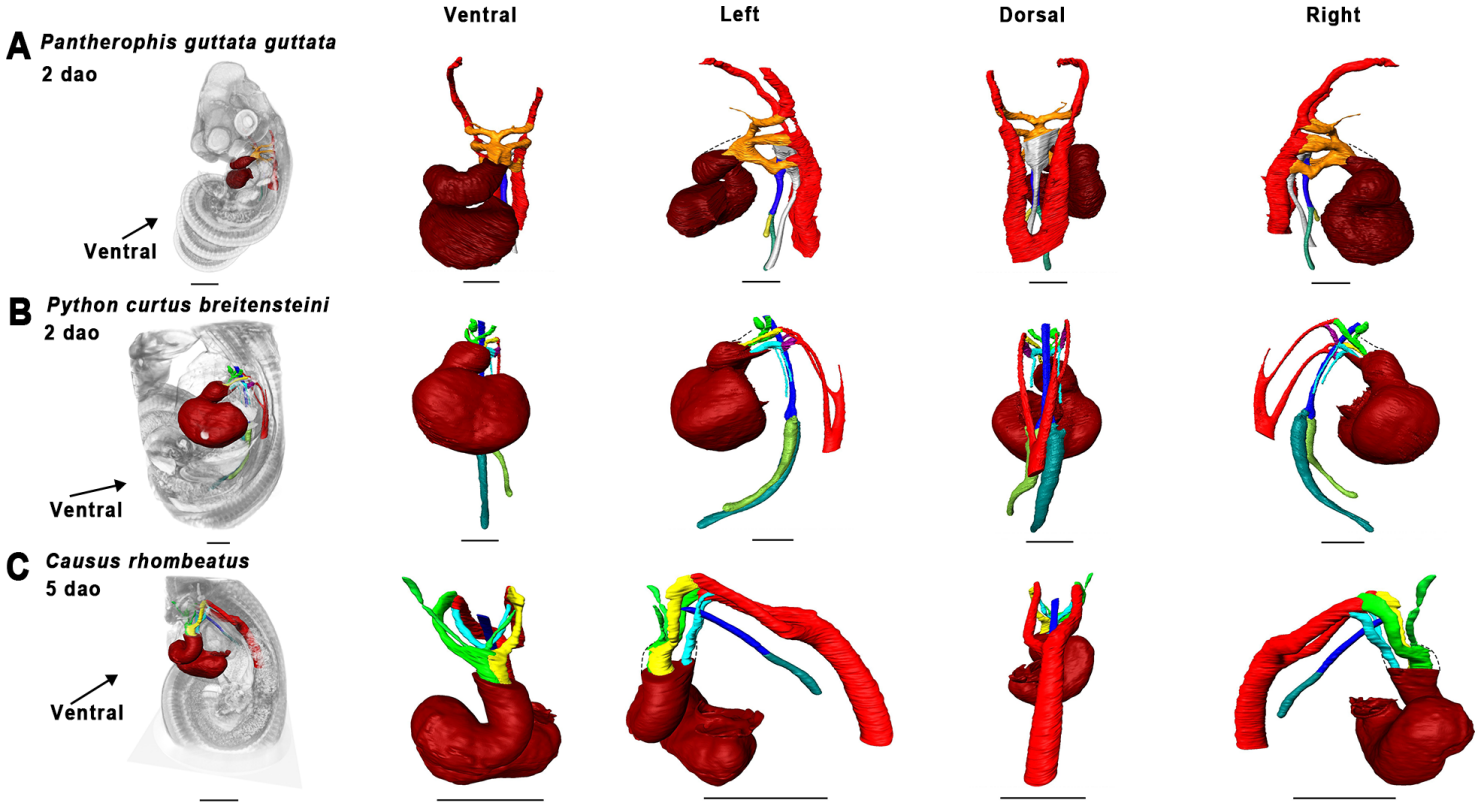
Overview of 3D reconstructions comparing similar developmental stages (Zehr 22–24 [40] **).** Visualized species are *Pantherophis guttata guttata* (A), *Python curtus breitensteini* (B) and *Causus rhombeatus* (C). Reconstructions were made in Amira 5.4.5. Dashed lines denote outflow tract contours. Scale bars represent 1 mm.

**Figure 14:**
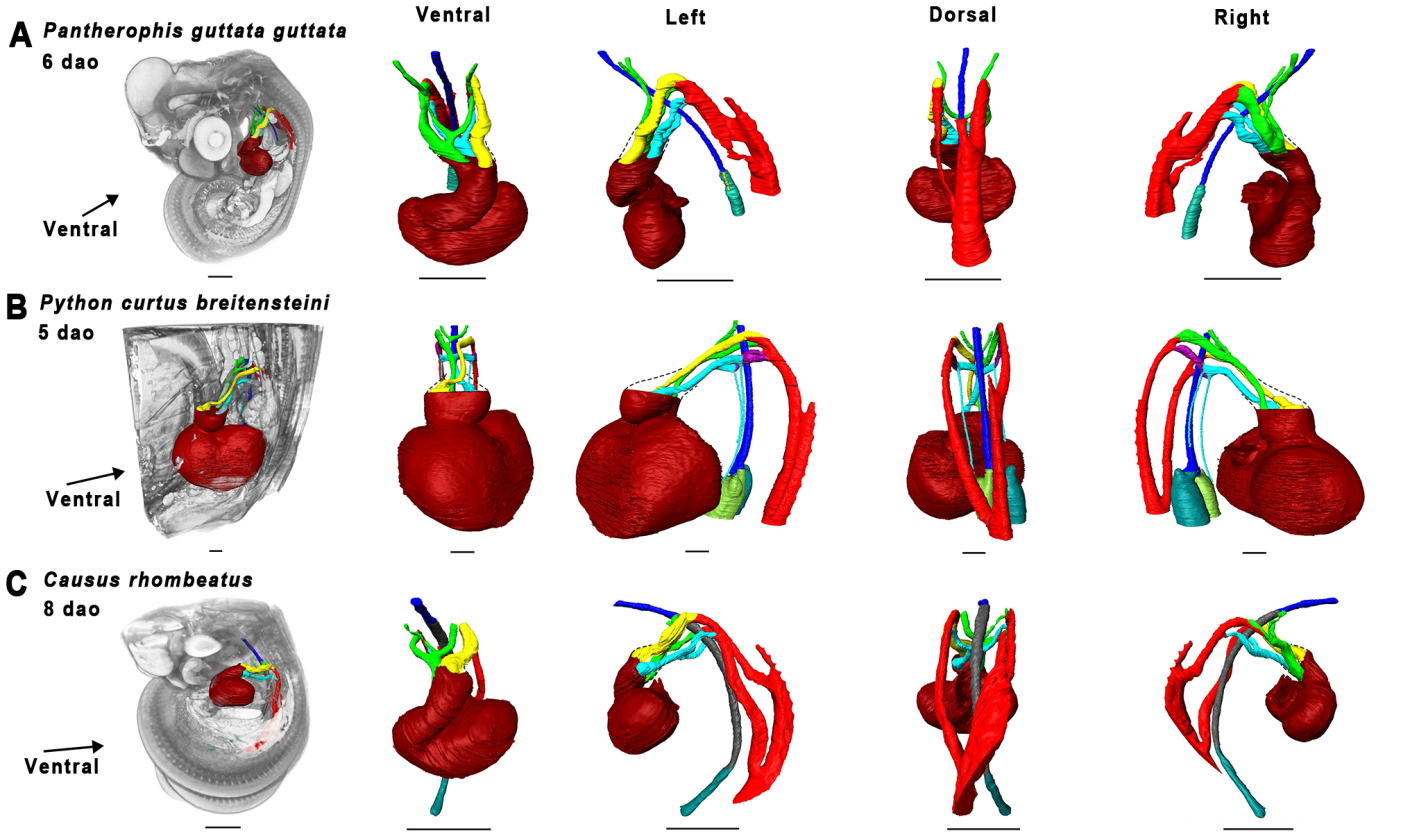
Overview of 3D reconstructions comparing similar developmental stages (Zehr 25–28 [40] **).** Visualized species are *Pantherophis guttata guttata* (A), *Python curtus breitensteini* (B) and *Causus rhombeatus* (C). Reconstructions were made in Amira 5.4.5. Dashed lines denote outflow tract contours. Scale bars represent 1 mm.

**Figure 15:**
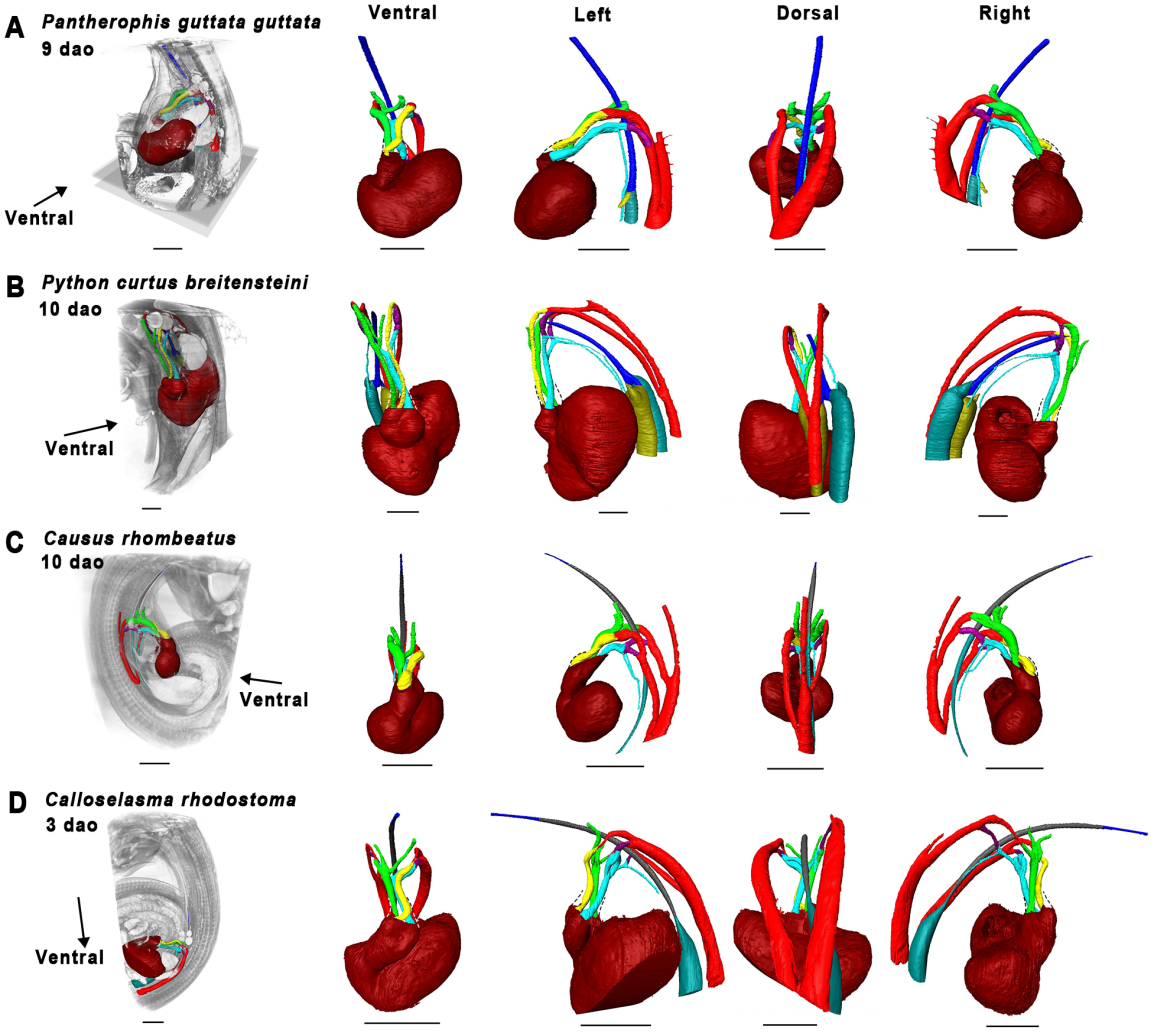
Overview of 3D reconstructions comparing similar developmental stages (Zehr 26–30 [40] **).** Visualized species are *Pantherophis guttata guttata* (A), *Python curtus breitensteini* (B), *Causus rhombeatus* (C) and *calloselasma rhodostoma* (D). Reconstructions were made in Amira 5.4.5.Dashed lines denote outflow tract contours. Scale bars represent 1 mm.

**Figure 16:**
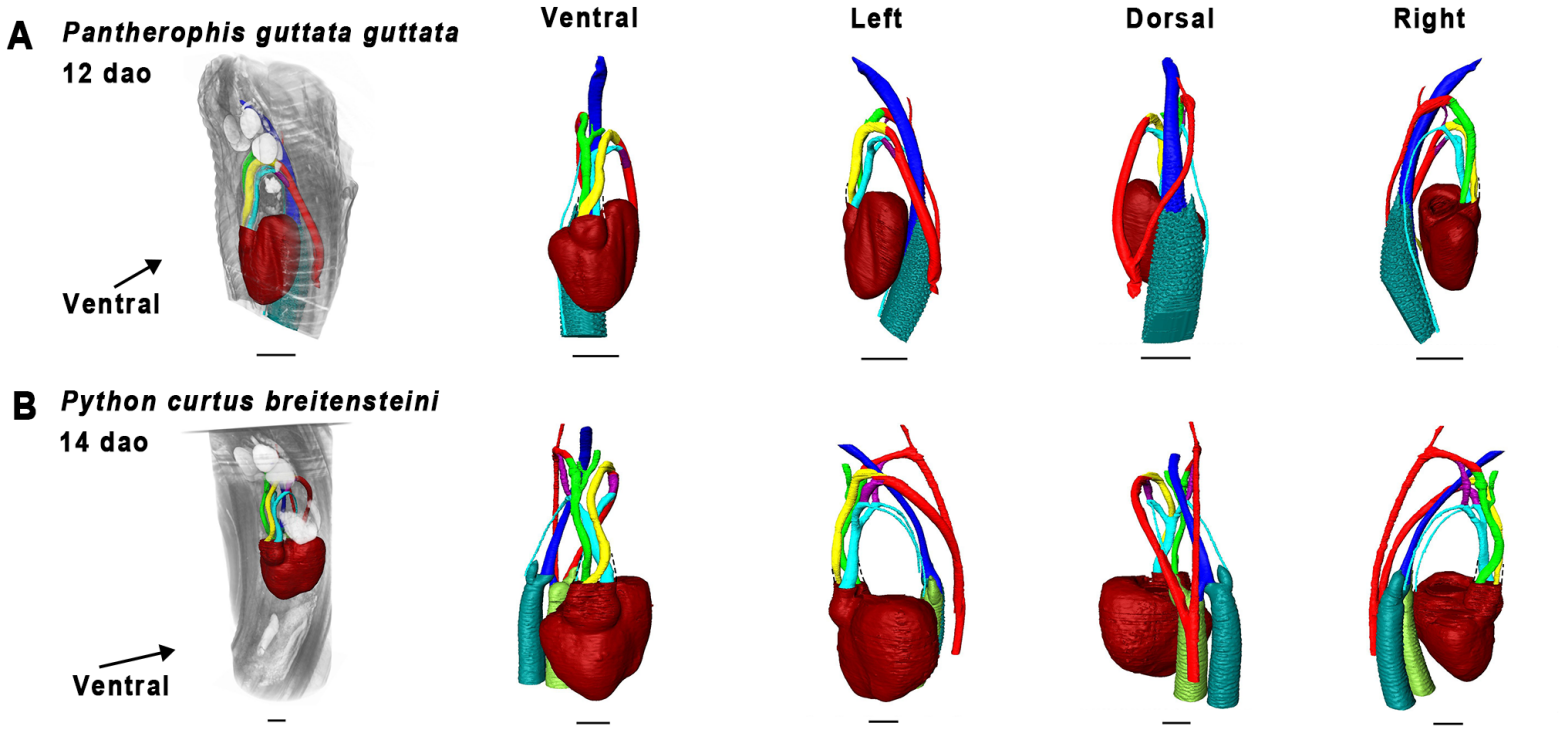
Overview of 3D reconstructions comparing similar developmental stages (Zehr 32–33 [40] **).** Visualized species are *Pantherophis guttata guttata* (A) and *Python curtus breitensteini* (B). Reconstructions were made in Amira 5.4.5. Dashed lines denote outflow tract contours. Scale bars represent 1 mm.

**Figure 17:**
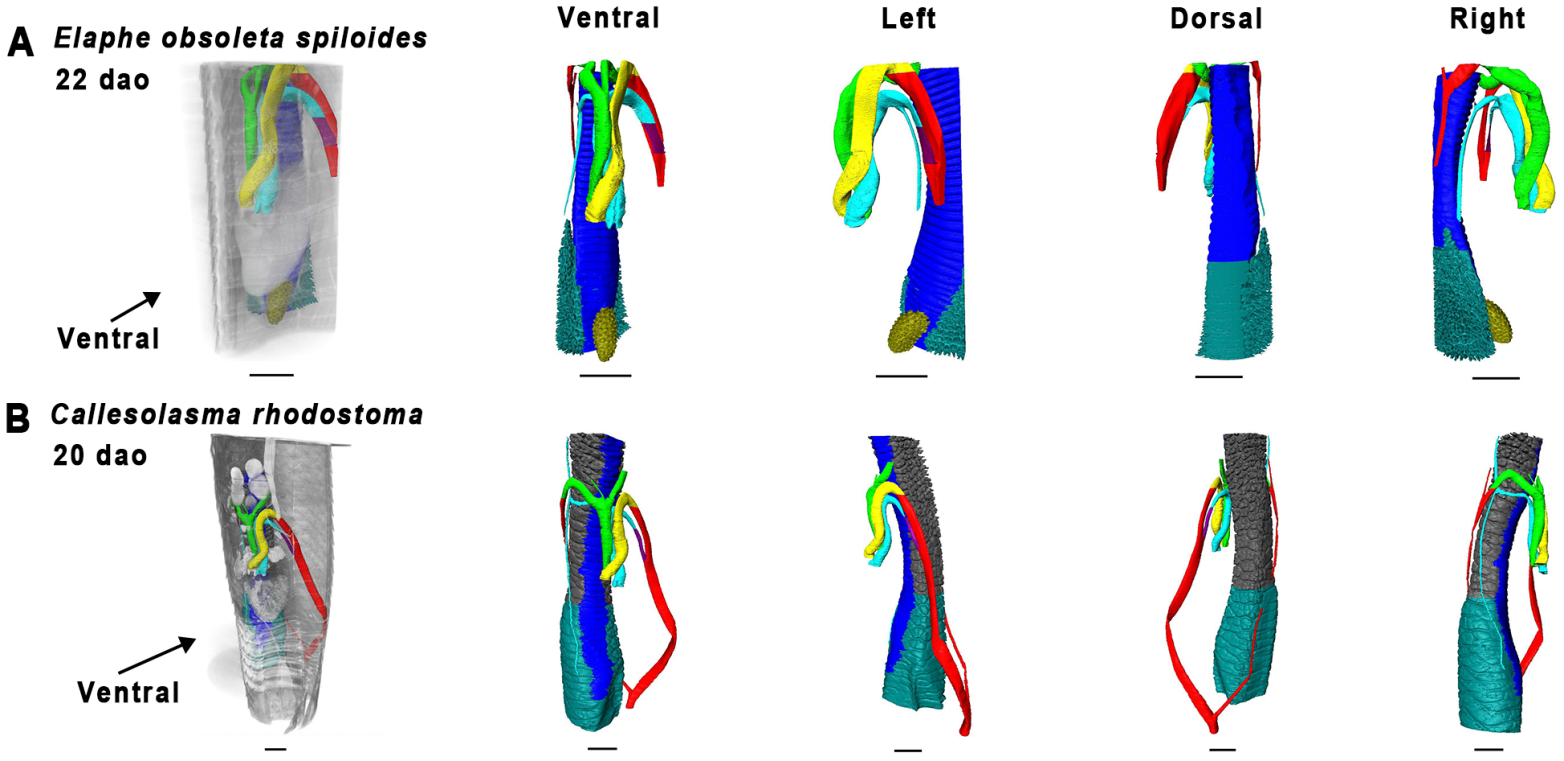
Overview of 3D reconstructions comparing similar developmental stages (Zehr 34–36 [40] **).** Visualized species are *Elaphe obsolete spiloides* (A), and *Calloselasma rhodostoma* (B). Reconstructions were made in Amira 5.4.5. Scale bars represent 1 mm.

Material used is listed in Table 3, and a legend to the color-coding of the Amira models is in Fig. 9. Figs. 10–12 show transverse sections just posterior to tracheal bifurcation or the tracheal lung of all specimens showing lung lumina. The Amira reconstructions can be viewed in Figs. 13–17. An overview of the developmental types described, cross-referenced with adult anatomies, is given in Table 5.

**Table 5:** Summary of some variations in the morphology of the lungs and pulmonary artery types in selected snake taxa (based on our own observations and Ref. [12]).

Development		Resulting lung pattern			Resulting PA* pattern			Taxa
Lung development type	Description of lung development type	Left lung	Right lung	Type [12]	Left PA	Right PA	Our type	Representative taxa
1	Right lung bud develops normally; left lung bud is absent. A tracheal lung may develop from the trachea.	Absent	Fully developed	1	Absent	Present	PA1 or PA2	Acrochordidae
2a	Right lung bud develops normally; left lung bud is present but fails to elongate and no faveoli are formed. A tracheal lung may develop from the trachea.	Vestigial, not faveolated	Fully developed	2	Absent	Present	PA1 or PA2	Colubridae
2b	Right lung bud develops normally; left lung bud is present but faveoli are formed. A tracheal lung may develop from the trachea.	Vestigial, faveolated	Fully developed	2	Minute, if present	Present	PA1 or PA2	Colubridae
3	Both right and left lung buds develop normally.	Fully developed	Fully developed	3	Present	Present	PA3	Boidae

*PA  =  Pulmonary Artery.

#### Zehr [40] stage 24–25

The extent of development of the heart and lungs varied considerably among our species sample at this stage. In *Pantherophis guttata guttata* (Fig. 13A), the tracheoesophageal septum had not formed, so that esophagus and trachea were still in continuity; this was not the case in the other species. In *P. guttata guttata* the right lung was longer than the left, but both had a distinct lumen distally (Fig. 10A). In *Python curtus breitensteini* (Fig. 13A) the right lung was longer than the left, but both featured a distinct lumen (Fig. 11A). In *Causus rhombeatus* (Fig. 12A; Fig. 13C) the left lung was absent.

*P. guttata guttata* (Fig. 13A) still had a common outflow tract. In *P. curtus breitensteini* (Fig. 13B) however, the outflow tract was divided into three channels: the left aorta, right aorta and the pulmonary channel. Finally, in *Causus rhombeatus* (Fig. 13C) only the aorticopulmonary septum had formed, dividing the outflow tract into a common aortic and a pulmonary channel. Regardless of developmental stage, the PAAs were in continuity with the two dorsal aortae, which originate from the ventricle. *P. curtus breitensteini* (Fig. 13B) was the only species to show two pulmonary arteries at this stage. Each artery extended to its respective lung from the sixth aortic arch; both ductus arteriosi were therefore distinct.

#### Zehr [40] stage 25–28

The esophagus and trachea in *P. guttata guttata* (Fig. 14A) were now separated by the trachea-esophageal septum. The right lung had elongated and acquired a distinct lumen in all species (Fig. 10B, Fig. 11B, Fig. 12B). The left lung in *P. curtus breitensteini* (Fig. 14B) was at a comparable stage of development to the right lung. By contrast, the left lung of *P. guttata guttata* (Fig. 14A) was smaller than the right and had only a small lumen distally, while the right also had distinct lumina at more anterior locations (Fig. 10B). In *Causus rhombeatus* (Fig. 14C) the tracheal lung could be identified for the first time as a thickening of the trachea at the level of the widening of the tracheal lumen. Posteriorly, the lumen widened more, denoting the start of the right lung (Fig. 12B; Fig. 14C).

With the establishment of the definitive left aorta, right aorta and pulmonary trunk, the vascular anatomy was now similar in all species. Except for *P. curtus breitensteini* (Fig. 14B), no pulmonary arteries had formed, although a layer of mesenchymal tissue (Fig. 10B) around the lungs in both *P. guttata guttata* and *Causus rhombeatus* might be interpreted as the splanchnic plexus from which the distal parts of the pulmonary arteries will develop.

#### Zehr [40] stage 26–30

The right lung had reached a similar stage of development in all species (Fig. 15A, B, C). In *P. guttata guttata* (Fig. 10C, Fig. 15A), no significant changes were observed. The left and right lungs in *P. curtus breitensteini* were equally developed (Fig. 15B). *Causus rhombeatus* and *Calloselasma rhodostoma* both had a tracheal lung (positioned anteriorly to the heart) and a right lung (Fig. 15C, D), but the right lung of *Calloselasma rhodostoma* was more obvious due to a constriction at cardiac level and luminal widening distally from this constriction (Fig. 12D); *Causus rhombeatus* lacked such a constriction and widening, a characteristic of a thoraconuchal lung (Fig. 12C).

At this stage, all species had one or two pulmonary arteries, arising posterior to the heart and connecting to their respective 6^th^ PAA, although their point of origin could not be found. So, the pulmonary artery originating at the right lung connects to right 6^th^ PAA. A left and right ductus arteriosus can be defined. At this developmental stage, the tracheal lung, where present, was not supplied by an obvious pulmonary artery; thus in both *Causus rhombeatus* (Fig. 15C) and *Calloselasma rhodostoma* (Fig. 15D) the pulmonary artery did not extend anterior to the heart. Possibly the tracheal lung was supplied with blood by a different vessel, or the anterior pulmonary artery was too small in diameter to be noticed or visualized reliably. If PFA did not bind with these thin vessels, it would not have appeared on the scans. Finally, *Causus rhombeatus* (Fig. 15C) had two pulmonary arteries that both lead to the single, right lung. Finally, *Calloselasma rhodostoma* (Fig. 15D) featured two left 6^th^ PAAs.

#### Zehr [40] stage 32–35

Specimens of *Calloselasma rhodostoma* or *Causus rhombeatus* were not available. Lung development in both *P. guttata guttata* (Fig. 16A) and *P. curtus breitensteini* (Fig. 16B) had reached a stage, at which separate faveoli became distinguishable. In *P. guttata guttata* (Fig. 16A) the left lung now featured a distinct lumen (Fig. 10D). Furthermore, the left lung was now in a more ventral location with respect to the right lung, compared with previous stages of this species examined.

Both *P. guttata guttata* (Fig. 16A) and *P. curtus breitensteini* (Fig. 16B) show changed spatial relations of the heart with respect to the PAAs: these are now located directly anterior to the heart. Furthermore, in *P. guttata guttata* (Fig. 16A) the right ductus arteriosus was becoming obliterated; in *P. curtus breitensteini*, the ductus was still apparent.

#### Zehr [40] stage 34–36

No specimen of *P. curtus breitensteini* was available. The lungs were in an advanced stage of development, with distinct faveoli and tracheal rings. In *Elaphe obsoleta spiloides* the left lung (Fig. 17A) featured distinct faveoli and a wide lumen (Fig. 10E). In *Calloselasma rhodostoma* (Fig. 17B) the faveoli of the tracheal lung had reached a state of maturity apparently similar to that of the right lung. It extended until approximately halfway along the ventricle, where a gradual widening of its lumen indicated the origin of the right lung (Fig. 12E), although there was no sharp boundary between the two.

The vascular anatomy of *E. obsoleta spiloides* (Fig. 17A) and *Calloselasma rhodostoma* (Fig. 17B) were highly similar. The right ductus arteriosus was completely obliterated, although in *E. obsoleta spiloides* remnants of the right 4^th^ and 6^th^ PAA could still be distinguished. On the left, the ductus arteriosus persisted. Differences were found in the pulmonary artery: in *Calloselasma rhodostoma* it curved anteriorly, not posteriorly as it does in *E. obsoleta spiloides*. Additionally, at the curvature where the single pulmonary artery curves anteriorly, a second pulmonary artery branched off in a posterior direction in *Calloselasma rhodostoma*.

## Discussion

### Classification of the pulmonary arteries in the adult snake

We have examined the adult anatomy of the pulmonary arteries in a broad phylogenetic sample of snake species. We find that the adult anatomical pattern of the pulmonary arteries in the species sampled can be described under three types, which we designate PA1-3. When examining published typifications [54]–[56], a common plan is revealed that corresponds to our type PA1. For example, Brongersma's types one and four [54] differ on a gross level only on the dorso-ventral position of the vessels. The other three Brongersma types can also be characterized as type PA1. This could be a consequence of the restricted species sample that Brongersma investigated: they are all Viperidae.

When we map our findings onto a phylogeny (Fig. 2), at least two shared primitive features in the macrostomata are recognized: the presence of an anteriorly extending pulmonary trunk and a right pulmonary artery, which can extend posteriorly or anteriorly. Of note is that *Hydrophis elegans* is the only snake with a PA type that is different from other Elapidae examined in this study. One possibility is that this is due to the lifestyle of this snake: *Hydrophis elegans* is the only purely aquatic snake [14] within the Elapidae examined by us; the others species are either terrestrial (*Bungarus candidus, Bungarus fasciatus*, *Lampropeltis triangulum sinaloea*, *Pantherophis guttata guttata* and *Elaphe obsoleta spiloides*) or semi-aquatic (*Natrix tesselata* and *Thamnophis sirtalis concinnus*).

Some species described in the literature but not sampled here are not easily reconciled with any one of our three types. These include *Agkistrodon contortrix mokasen* [57], *Charina bottae* [10], *Crotalus atrox* [56], *Crotalus horridus* [56], *Crotalus viridis* [56], *Elaphe obsoleta quadrivittata* [10], *Python regius* [58], [59] Family Pareatidae [60], *Sistrurus ravus* [56], and Family Typhlopidae [61]. It is not clear whether this is due to differences in interpretation, or to our limited sample size. Alternative nomenclatures, such as that used by Robb [61] for the Typhlopidae, will lead to different interpretations even if the pulmonary artery anatomy is the same.

### Development of lungs and pulmonary arteries

We show that the development of lungs and the surroundings splanchnic plexus [25], [30], [31], [33], [62], which differentiate into the pulmonary arteries, is a tightly integrated process, in which some deviations from a common plan appear early. Therefore, if the left lung bud fails to develop the splanchnic plexus will not develop either. As a result, the left pulmonary artery will also not develop.

#### Development of the right lung

The right bronchial bud is established early in development and develops into a mature right lung in all species. *Causus rhombeatus* deviates from the common plan in that it has a thoraconuchal lung in which the right lung and the tracheal lung are continuous. Already at early stages of tracheal lung development it was hard to distinguish it from the right lung. Thus, the question arises to what extent the development of right and tracheal lung are integrated. Further studies into the development of the thoraconuchal lung would therefore be of interest.

In *Calloselasma rhodostoma* the transition point from tracheal to right lung is demarcated by a constriction in the tracheal lumen at the axial level of the heart. However, this constriction disappeared at later stages of development. Regardless of the mode of development of the right lung, the right pulmonary artery always developed and became connected with the right branch of the 6^th^ PAA. As a result, this branch always persisted to some extent, as in *Calloselasma rhodostoma*, *E. obsolete spiloides* and *P. guttata guttata* the right ductus arteriosus regressed. In *P. curtus breitensteini* we lacked developmental stages sufficiently advanced to provide more definitive conclusions.

#### Development of the left lung

Our data show that four developmental patterns of the left lung can be distinguished. The first (Table 5, Type 1) is characterized by absence of a left bronchial bud in both *Calloselasma rhodostoma* and *Causus rhombeatus*. However, we lacked the youngest of embryonic stages, so that we could not determine whether a left bronchial bud does not develop due to absence of tracheal bifurcation, or whether it regresses shortly after its formation. Both developmental patterns result in type 1 lung anatomy (Table 5).

The second and third developmental patterns (Table 5, Type 2a and 2b) were observed in snakes with a vestigial left lung. This condition implies inhibition of lung elongation, but not necessarily of lung maturation: in the majority of species the vestigial left lung is faveolated and vascularized [12], which are typical anatomical features of a mature lung. Moreover, a vascularized vestigial left lung may be served by a minute pulmonary artery [12], which indicates a contribution to respiration. We did not observe any such minute pulmonary artery in our 22 dao *E. obsolete spiloides* specimen, but it is possible that the resolution of the MicroCT scans was either insufficient to detect this artery, or its presence was obscured by nearby structures. In the snakes that possess a vestigial left lung, it is usually both faveolated and vascularised [12].

Based on the observation that the vestigial left lung may or may not be faveolated and vascularized, the vestigial nature of the left lung may result from two processes: inhibition of early elongation of the left bronchial bud, and inhibition of later faveolization and vascularization. Thus we are able to identify two developmental patterns. In both cases the left bronchial bud undergoes significant slowing shortly after appearing, followed by arrest of elongation, and then either (1) fails to become vascularized (Table 5, Type 2a), (2) or undergoes subsequent vascularization (Table 5, Type 2b). Both development patterns result in type 2 lung anatomy (Table 5).

The fourth developmental pattern (Table 5, Type 3) leads to a fully developed, albeit relatively short left lung. The question arises whether the left lung is shorter than the right lung because it develops slower, as suggested previously [15], [63], or because its growth is arrested early (truncation). The earliest stage of *Python curtus breitensteini* in our sample (Fig. 13B) shows that the left lung is shorter than the right lung. Since already at this early stage the two lungs in this species are subequal, our data are most easily explained, in view of previous studies in *Thamnophis radix* and *Natrix natrix* [15], [24], by assuming that the left lung has a lower growth rate than the right lung, as opposed to truncation at a later stage of development. It is important to note that availability of this rare embryonic material is limited, and so it was not possible to make any quantitative determination of growth rates. It is also possible that the left lung bud initiated its development at a later stage (also a kind of heterochrony) or was smaller from the beginning (perhaps due to differences in cell allocation between the left and right buds). Further studies on a larger series of python embryos is needed to examine these issues.

To summarize, we propose that a stepwise change in developmental mechanisms resulted in the obliteration of the left lung. First, in basal macrostomata (represented by *Python curtus breitensteini* in this study), slowing of left lung elongation leads to a left lung that is shorter than the right lung. Secondly, in more advanced snakes (represented by *Pantherophis guttata guttata* and *Elaphe obsolete spiloides*), the arrest of elongation (truncation) later in left lung bud development results in a vestigial, faveolated left lung. Finally, failure to faveolate results in a vestigial left lung lacking faveoli. See Fig. 2 for a mapping of these events onto our phylogeny.

#### Development of the tracheal lung

Our results suggest that the tracheal lung arises as an outgrowth of respiratory tissue from the trachea. In the adult, the tracheal rings are open on the dorsal side so that the lumen of the tracheal lung and trachea communicate. Since tracheal rings are typically C-shaped [12], it is possible that respiratory tissue evaginates from the dorsal cleft in the trachea. A follow-up study investigating the specifics of tracheal lung development would be of great value. Tbx5 gene expression studies could be of special interest, given its role in formation of the tracheal cartilage in mice [5].

We show that no distinct pulmonary artery develops in relation to the tracheal lung; instead it might be supplied by the tracheal artery, which in humans branches of the inferior thyroid artery [64]. In snakes, however, it usually branches of the carotid artery [65]–[70], which does not correlate with the anterior pulmonary artery described here and in the literature (S1 Table). We, therefore, suggest that the anterior pulmonary artery is either an anteriorly relocated posterior pulmonary artery (as we propose for *Calloselasma rhodostoma*), or a branch of a posterior pulmonary artery (as we propose for *Hydrophis elegans*).

The tracheal lung and associated pulmonary artery were previously proposed to be an anteriorly relocated left lung and pulmonary artery, respectively [61]. This was disproved [71] when the presence of a ligamentum arteriosum between pulmonary trunk and left aorta was highlighted in snakes lacking a left pulmonary artery, which agrees with our results; the anterior pulmonary artery can therefore not be analogous with the left pulmonary artery.

We here propose a new hypothesis: descent of the heart, cephalic folding and elongation of the neck during development cause a change in pulmonary artery location with respect to the heart. While the lungs remain in a relatively stable location, the heart will pass the location where the pulmonary arteries attach to the lung. An embryonic posterior pulmonary artery will thus become relocated to an anterior position. Our observation in 20 dao *Calloselasma rhodostoma* is thus easily explained. In this species, the pulmonary artery curved posteriorly in early stages, but anteriorly in late stages. Additionally, the posterior branch was but a branch off the anteriorly curving pulmonary artery. This resembles our dissected *Trimeresurus* heart, and the same applies to *Causus rhombeatus*, whose adults are reported to exhibit two anterior pulmonary arteries [54]: it is a flipped version of the embryonic pulmonary artery anatomy found in this study.

Some snakes however, with have respiratory tissue along the entire longitudinal axis of the body [14], will have respiratory tissue posterior to the heart even after the descent of the heart, such as *Hydrophis* (Fig. 8). In this species the primary pulmonary artery in this species is likely not the anterior one but the posterior one. Further research into the development of such secondary pulmonary arteries is warranted.

## Conclusion

We have studied the development of the lungs and pulmonary arteries in snakes. We conclude that right and tracheal lung each develops via a common plan across species. By contrast, the left lung bud may (1) never develop; (2) become arrested after tracheal bifurcation, either without (2a) or with (2b) subsequent development of faveoli and vascularisation; (3) or elongate and differentiate normally, but at a slower rate than the right. The pulmonary arteries develop from locations posterior to the heart, but only if the relevant lung is functionally significant. No pulmonary artery develops in relation to the tracheal lung. Instead we propose that descent of the heart down the body, cephalic folding and elongation of the neck cause the heart to shift to a more posterior location with respect to the lung. A right posterior pulmonary artery may thus become a right anterior pulmonary artery.

This study shows that asymmetry in the snake respiratory system manifests itself early in development. This is in accordance with an earlier study [15]. It therefore seems likely that lung asymmetry appeared in the common ancestor to the serpentes, as opposed to having evolved independently several times. This suggests that the ancestral condition was one of pulmonary symmetry, as suggested by Kardong [10]. Reconstruction of further evolutionary events leading to the current asymmetrical pattern in the snake respiratory system requires more data. We propose that the left lung was gradually obliterated due to an in series placement of several developmental mechanisms: slowed elongation, truncation and failure to faveolate. However, it is also possible that, from the ancestral condition, the right lung has elongated and the ancestral length of the left lung persisted. It is also possible that a combination of these events took place.

We recommend that future work be aimed at exploring the development of additional snake species, and elucidating the genetic mechanisms influencing the various left and right lung growth rates. These should then be compared with developmental mechanisms of the lungs in relevant outgroups (such as *Anolis* or *Pogona*). Studies in mouse suggest that regulatory genes such as Tbx4, Tbx5 and Pitx2 are involved in patterning of the lungs, and thus the manifestation of asymmetry. However, left and right lung growth speeds might be mediated independently from each other by Shh [72] and sprouty [73] (see for reviews Refs. [1], [2], [7], [74]). Using the data provided by this study on the timing of developmental events in the snake respiratory system, it has become possible to study the involvement of these genes in the development of the snake respiratory system.

## Supporting Information

S1 Table **Overview of all literature dealing with cardiopulmonary connections known to us, including other observations (if available).** Note that the data on the direction and number of cardiopulmonary connection vessels have been simplified; extensive detail of the original literature made it hard to summarize the data otherwise. L Type  =  lung type (based on [12]), nPA  =  number of pulmonary arteries, Direct. PA  =  direction of the pulmonary artery, nPV  =  number of pulmonary veins, Direct PV  =  direction of the pulmonary vein, Heart pos.  =  Heart position along long body axis.
(DOC)
