# Supplementary material for: Heterochrony and Early Left-Right Asymmetry in the Development of the Cardiorespiratory System of Snakes
Source: PLoS One. 2015 Jan 2;10(1):e116416. doi: 10.1371/journal.pone.0116416 (PMC4282204; doi:10.1371/journal.pone.0116416)
Supplement: S1 Table — Overview of all literature dealing with cardiopulmonary connections known to us, including other observations (if available). Note that the data on the direction and number of cardiopulmonary connection vessels have been simplified; extensive detail of the original literature made it hard to summarize the data otherwise. L Type = lung type (based on [12]), nPA = number of pulmonary arteries, Direct. PA = direction of the pulmonary artery, nPV = number of pulmonary veins, Direct PV = direction of the pulmonary vein, Heart pos. = Heart position along long body axis. (DOC) [file pone.0116416.s001.doc]

Table S1: Overview of all literature dealing with snake pulmonary arteries known to us. Note that the data on the direction and number of pulmonary arteries have been simplified to our typification (Figure 4). LType = lung type (based on [1]), nPA = number of pulmonary arteries, Direct. PA = direction of the pulmonary artery, nPV = number of pulmonary veins, Direct PV = direction of the pulmonary vein, Heart pos. = Heart position along long body axis.

| **Species** | **Species as mentioned in source** | **Family** | **Source** | **LType** | **nPA** | **Direct. PA** | **nPV** | **Direct. PV** | **Heart pos.** | **Brongersma (1949) type** | **Author's type** |
| --- | --- | --- | --- | --- | --- | --- | --- | --- | --- | --- | --- |
| *Acrochordus granulatus* | *Acrochordus granulatus* | Acrochordidae | [2] | Type 1 | 2 | both | 2 | both | central? |  | PA1 |
| *Acrochordus granulatus* | *Acrochordus granulatus* | Acrochordidae | [3] | Type 2 | 2 | both | 2 | both | central |  | PA1 |
| *Anilius scytale* | *Anilius scytale* | Anilidae | [4] | Type 3 | 2 | posterior | 2 | anterior | anterior |  | PA3 |
| *Cylindrophis boulengeri* | *Cylindrophis boulengeri* | Anilidae | [4] | Type 3 | 2 | posterior | 2 | anterior | anterior |  | PA3 |
| *Cylindrophis isolepis* | *Cylindrophis isolepis* | Anilidae | [4] | Type 3 | 2 | posterior | 2 | anterior | anterior |  | PA3 |
| *Cylindrophis masculatus* | *Cylindrophis masculatus* | Anilidae | [4] | Type 3 | 2 | posterior | 2 | anterior | anterior |  | PA3 |
| *Cylindrophis rufus* | *Cylindrophis rufus* | Anilidae | [4] | Type 3 | 2 | posterior | 2 | anterior | anterior |  | PA3 |
| *Boa constrictor constrictor* | *Constrictor constrictor constrictor* | Boidae | [4] | Type 3 | 2 | posterior | 2 | anterior | anterior |  | PA3 |
| *Boa constrictor imperator* | *Constrictor constrictor imperator* | Boidae | [4] | Type 3 | 2 | posterior | 2 | anterior | anterior |  | PA3 |
| *Boa constrictor occidentalis* | *Constrictor constrictor occidentalis* | Boidae | [4] | Type 3 | 2 | posterior | 2 | anterior | anterior |  | PA3 |
| *Boa enydris enydris* | *Boa enydris enydris* | Boidae | [4] | Type 3 | 2 | posterior | 2 | anterior | anterior |  | PA3 |
| *Boa madagascariensis* | *Acrantophis madagascariensis* | Boidae | [4] | Type 3 | 2 | posterior | 2 | anterior | anterior |  | PA3 |
| *Boa manditra* | *Sanzinia madagascariensis* | Boidae | [4] | Type 3 | 2 | posterior | 2 | anterior | anterior |  | PA3 |
| *Bothrochilus boa* | *Bothrochilus boa* | Boidae | [4] | Type 3 | 2 | posterior | 2 | anterior | anterior |  | PA3 |
| *Charina bottae* | *Charina bottae* | Boidae | [5] | Type 3 | 2 | posterior | 1 | anterior | posterior |  | PA3 |
| *Charina bottae bottae* | *Charina bottae bottae* | Boidae | [4] | Type 3 | 2 | posterior | 2 | anterior | anterior |  | PA3 |
| *Charina reinhardtii* | *Calabaria reinhardtii* | Boidae | [4] | Type 3 | 2 | posterior | 2 | anterior | anterior |  | PA3 |
| *Corallus caninus* | *Boa canina* | Boidae | [4] | Type 3 | 2 | posterior | 2 | anterior | anterior |  | PA3 |
| *Enygrus asper schmidti* | *Enygrus asper schmidti* | Boidae | [4] | Type 3 | 2 | posterior | 2 | anterior | anterior |  | PA3 |
| *Enygrus bibroni bibroni* | *Enygrus bibroni bibroni* | Boidae | [4] | Type 3 | 2 | posterior | 2 | anterior | anterior |  | PA3 |
| *Enygrus carinatus* | *Enygrus carinatus* | Boidae | [4] | Type 3 | 2 | posterior | 2 | anterior | anterior |  | PA3 |
| *Epicrates cenchria cenchria* | *Epicrates cenchria cenchria* | Boidae | [4] | Type 3 | 2 | posterior | 2 | anterior | anterior |  | PA3 |
| *Epicrates inornatus inornatus* | *Epicrates inornatus inornatus* | Boidae | [4] | Type 3 | 2 | posterior | 2 | anterior | anterior |  | PA3 |
| *Epicrates striatus striatus* | *Epicrates striatus striatus* | Boidae | [4] | Type 3 | 2 | posterior | 2 | anterior | anterior |  | PA3 |
| *Eryx jaculus jaculus* | *Eryx jaculus jaculus* | Boidae | [4] | Type 3 | 2 | posterior | 2 | anterior | anterior |  | PA3 |
| *Eryx johnii johnii* | *Eryx johnii johnii* | Boidae | [4] | Type 3 | 2 | posterior | 2 | anterior | anterior |  | PA3 |
| *Eunectes murinus* | *Eunectes scytale* | Boidae | [4] | Type 3 | 2 | posterior | 2 | anterior | anterior |  | PA3 |
| *Liasis amethistinus* | *Liasis amethistinus amethistinus* | Boidae | [4] | Type 3 | 2 | posterior | 2 | anterior | anterior |  | PA3 |
| *Liasis amethistinus kinghorni* | *Liasis amethistinus kinghorni* | Boidae | [4] | Type 3 | 2 | posterior | 2 | anterior | anterior |  | PA3 |
| *Liasis fuscus albertisii* | *Liasis fuscus albertisii* | Boidae | [4] | Type 3 | 2 | posterior | 2 | anterior | anterior |  | PA3 |
| *Liasis mackloti mackloti* | *Liasis mackloti mackloti* | Boidae | [4] | Type 3 | 2 | posterior | 2 | anterior | anterior |  | PA3 |
| *Liasis olivaceus papuanus* | *Liasis olivaceus papuanus* | Boidae | [4] | Type 3 | 2 | posterior | 2 | anterior | anterior |  | PA3 |
| *Lichanura roseofusca roseofusca* | *Lichanura roseofusca roseofusca* | Boidae | [4] | Type 3 | 2 | posterior | 2 | anterior | anterior |  | PA3 |
| *Morelia spilota* | *Morelia argus* | Boidae | [4] | Type 3 | 2 | posterior | 2 | anterior | anterior |  | PA3 |
| *Morelia viridis* | *Chondropython viridis* | Boidae | [4] | Type 3 | 2 | posterior | 2 | anterior | anterior |  | PA3 |
| *Python curtus brongersmai* | *Python curtus brongersmai* | Boidae | [4] | Type 3 | 2 | posterior | 2 | anterior | anterior |  | PA3 |
| *Python curtus curtus* | *Python curtus curtus* | Boidae | [4] | Type 3 | 2 | posterior | 2 | anterior | anterior |  | PA3 |
| *Python molurus bivittatus* | *Python molurus bivittatus* | Boidae | [4] | Type 3 | 2 | posterior | 2 | anterior | anterior |  | PA3 |
| *Python molurus molurus* | *Python molurus molurus* | Boidae | [4] | Type 3 | 2 | posterior | 2 | anterior | anterior |  | PA3 |
| *Python regius* | *Python regius* | Boidae | [4] | Type 3 | 2 | posterior | 2 | anterior | anterior |  | PA3 |
| *Python regius* | *Python regius* | Boidae | [6] | ? | 1 | posterior | 1 | anterior | ? |  | PA2 |
| *Python regius* | *Python regius* | Boidae | [7] | Type 3 | 2 | posterior | 2 | anterior | anterior |  | PA3 |
| *Python regius* | *Python regius* | Boidae | [8] | Type 3 | 2 | posterior | 1 | anterior | posterior |  | PA3 |
| *Python reticulatus* | *Boa reituclata* | Boidae | [9] | Type 3 | 2 | posterior | 2 | anterior | anterior |  | PA3 |
| *Python reticulatus* | *Python reticulatus* | Boidae | [4] | Type 3 | 2 | posterior | 2 | anterior | anterior |  | PA3 |
| *Python sebae* | *Python sebae* | Boidae | [4] | Type 3 | 2 | posterior | 2 | anterior | anterior |  | PA3 |
| *Python timoriensis* | *Python timoriensis* | Boidae | [4] | Type 3 | 2 | posterior | 2 | anterior | anterior |  | PA3 |
| *Trachyboa gularis* | *Trachyboa gularis* | Boidae | [10] | Type 1/Type 2 | 2 | both | 2 | both | central? |  | PA1 |
| *Tropidophis maculatus haetianus* | *Tropidophis maculatus haetianus* | Boidae | [10] | Type 1/Type 2 | 2 | both | ? | ? | central? |  | PA1 |
| *Tropidophis melanurus* | *Tropidophis melanurus* | Boidae | [10] | Type 1/Type 2 | 2 | both | ? | ? | central? |  | PA1 |
| *Tropidophis pardalis pardalis* | *Tropidophis pardalis pardalis* | Boidae | [10] | Type 1/Type 2 | 2 | both | ? | ? | central? |  | PA1 |
| *Coluber constrictor* | *Zamenis constrictor* | Colubridae | [11] | Type 1 | 1 | posterior | 1 | anterior | anterior |  | PA2 |
| *Dipsas albifrons* | *Dipsas albifrons* | Colubridae | [12] | Type 1/Type 2 | 2 | both | ? | ? | central |  | PA1 |
| *Dipsas caresbyi* | *Dipsas caresbyi* | Colubridae | [12] | Type 1/Type 2 | 2 | both | ? | ? | central |  | PA1 |
| *Dipsas indica* | *Dipsas indica* | Colubridae | [12] | Type 1/Type 2 | 2 | both | ? | ? | central |  | PA1 |
| *Dipsas mikanii* | *Dipsas mikanii* | Colubridae | [12] | Type 1/Type 2 | 2 | both | ? | ? | central |  | PA1 |
| *Dipsas pavonina* | *Dipsas pavonina* | Colubridae | [12] | Type 1/Type 2 | 2 | both | ? | ? | central |  | PA1 |
| *Dipsas variegata* | *Dipsas variegata* | Colubridae | [12] | Type 1/Type 2 | 2 | both | ? | ? | central |  | PA1 |
| *Elaphe flavolineata* | *Elaphe flavolineata* | Colubridae | [4] | Type 2? | 1 | posterior | 1 | anterior | anterior |  | PA3 |
| *Elaphe obsoleta quadrivittata* | *Elaphe obsoleta quadrivittata* | Colubridae | [13] | Type 1/Type 2 | 1 | posterior | 1 | anterior | ? |  | PA2 |
| *Elaphe radiata* | *Elaphe radiata* | Colubridae | [4] | Type 2 | 1 | posterior | 1 | anterior | anterior |  | PA3 |
| *Erythrolamprus aesculapii* | *Erythrolamprus aesculapii* | Colubridae | [14] | Type 2? | 2 | both | 2 | both | central |  | PA1 |
| *Natrix natrix* | *Tropidonotus natrix* | Colubridae | [15] | Type 1 | 1 | posterior | 1 | anterior | ? |  | PA2 |
| *Elaphe obsoleta quadrivittata* | *Elaphe obsoleta quadrivittata* | Colubridae | [5] | Type 1/Type 2 | 1 | posterior | 2? | both | ? |  | PA2 |
| *Ptyas mucosus* | *Ptyas mucosus* | Colubridae | [16,17] | Type 1/Type 2 | 1 | posterior | 1 | anterior | anterior |  | PA2 |
| *Thamnophis sirtalis* | *Thamnophis sirtalis* | Colubridae | [18] | Type 2 | 1 | posterior | 1 | anterior | anterior |  | PA2 |
| *Bungarus candidus* | *Bungarus candidus* | Elapidae | [4] | Type 2 | 1 | posterior | 1 | anterior | anterior |  | PA3 |
| *Bungarus fasciatus* | *Bungarus fasciatus* | Elapidae | [4] | Type 2 | 1 | posterior | 1 | anterior | anterior |  | PA3 |
| *Naja naja* | *Naja naja* | Elapidae | [19] | ? | ? | ? | 1 | anterior | ? |  | PV3 |
| *Naja naja naja* | *Naja naja naja* | Elapidae | [20] | Type 1 | 1 | both | ? | ? | central |  | PA2 |
| *Aplopeltura boa* | *Aplopeltura boa* | Pareatidae | [12] | Type 1/Type 2 | 1 | posterior | ? | ? | posterior? |  | PA2 |
| *Pareas carinatus* | *Pareas carinatus* | Pareatidae | [12] | Type 2 | 2 | both? | ? | ? | central? |  | PA1 |
| *Pareas hamptoni* | *Pareas hamptoni* | Pareatidae | [12] | Type 1/Type 2 | 1? | posterior? | ? | ? | anterior? |  | PA2? |
| *Pareas laevis* | *Pareas laevis* | Pareatidae | [12] | Type 1 | 1 | posterior | ? | ? | posterior? |  | PA2 |
| *Pareas malaccanus* | *Pareas malaccanus* | Pareatidae | [12] | Type 1/Type 2 | 1? | posterior? | ? | ? | anterior? |  | PA2? |
| *Pareas margatirophorus* | *Pareas margatirophorus* | Pareatidae | [12] | Type 1/Type 2 | 1? | posterior? | ? | ? | anterior? |  | PA2? |
| *Pareas nuchalis* | *Pareas nuchalis* | Pareatidae | [12] | Type 1/Type 2 | 1? | posterior? | ? | ? | anterior? |  | PA2? |
| *Pareas vertebralis* | *Pareas vertebralis* | Pareatidae | [12] | Type 1/Type 2 | 2 | both | ? | ? | central |  | PA1 |
| *Ramphotyphlops australis* | *Typhlops australis* | Typhlopidae | [21] | Type 1/Type 2 | 2 | both | 2 | both | central |  | PA1 |
| *Ramphotyphlops ligatus* | *Typhlops ligatus* | Typhlopidae | [21] | Type 1/Type 2 | 2 | both | 2 | both | central |  | PA1 |
| *Ramphotyphlops polygrammicus* | *Typhlops polygrammicus* | Typhlopidae | [21] | Type 1/Type 2 | 2 | both | 2 | both | central |  | PA1 |
| *Ramphotyphlops proximus* | *Typhlops proximus* | Typhlopidae | [21] | Type 1/Type 2 | 2 | both | 2 | both | central |  | PA1 |
| *Rhinotyphlops acutus* | *Typhlops acutus* | Typhlopidae | [22] | Type 1/Type 2 | 2 | both | 2 | both | central |  | PA1 |
| *Agkistrodon agkistrodon* | *Agkistrodon agkistrodon* | Viperidae | [23] | Type 2 | 2 | both | ? | ? | central | IIIA | PA1 |
| *Agkistrodon contortrix mokasen* | *Agkistrodon Contortrix mokeson* | Viperidae | [24] | Type 1 | 1 | anterior | ? | ? | central? |  | PA2? |
| *Agkistrodon piscivorus* | *Agkistrodon piscivorus* | Viperidae | [25] | Type 1/Type 2 | 2 | both | ? | ? | central | III | PA1 |
| *Agkistrodon piscivorus leucostoma* | *Agkistrodon piscivorus leucostoma* | Viperidae | [23] | Type 2 | 2 | both | ? | ? | central | IIIA | PA1 |
| *Agkistrodon piscivorus piscivorus* | *Agkistrodon piscivorus piscivorus* | Viperidae | [23] | Type 2 | 2 | both | ? | ? | central | IIIA | PA1 |
| *Atheris chlorechis* | *Atheris chlorechis* | Viperidae | [25] | Type 1/Type 2 | 2 | both | ? | ? | central | III | PA1 |
| *Atractaspis bibronii* | *Atractaspis bibronii* | Viperidae | [25] | Type 1/Type 2 | 2 | both | ? | ? | central | IV | PA1 |
| *Atractaspis irregularis* | *Atractaspis irregularis* | Viperidae | [25] | Type 1/Type 2 | 2 | both | ? | ? | central | IV | PA1 |
| *Atropoides nummifer* | *Bothrops nummifer* | Viperidae | [25] | Type 1/Type 2 | 2 | both | ? | ? | central | III | PA1 |
| *Bitis arietans* | *Bitis arictans* | Viperidae | [25] | Type 1/Type 2 | 2 | both | ? | ? | central | I | PA1 |
| *Bitis gabonica* | *Bitis gabonica* | Viperidae | [25] | Type 1/Type 2 | 2 | both | ? | ? | central | I | PA1 |
| *Bitis nasicornis* | *Bitis nasicornis* | Viperidae | [26] | Type 1 | 2 | both | 2 | both | central |  | PA1 |
| *Bothriechis schlegelii* | *Bothrops schlegelii* | Viperidae | [25] | Type 1/Type 2 | 2 | both | ? | ? | central | III | PA1 |
| *Bothrops alternatus* | *Bothrops alternatus* | Viperidae | [25] | Type 1/Type 2 | 2 | both | ? | ? | central | I | PA1 |
| *Bothrops ammodytoides* | *Bothrops ammodytoides* | Viperidae | [25] | Type 1/Type 2 | 2 | both | ? | ? | central | I | PA1 |
| *Bothrops atrox* | *Bothrops atrox* | Viperidae | [25] | Type 1/Type 2 | 2 | both | ? | ? | central | I | PA1 |
| *Bothrops bilineatus* | *Bothrops bilincatus* | Viperidae | [25] | Type 1/Type 2 | 2 | both | ? | ? | central | I | PA1 |
| *Bothrops jararacussu* | *Bothrops jararacussu* | Viperidae | [25] | Type 1/Type 2 | 2 | both | ? | ? | central | I | PA1 |
| *Bothrops lansbergii* | *Bothrops lansbergii* | Viperidae | [25] | Type 1/Type 2 | 2 | both | ? | ? | central | III | PA1 |
| *Calloselasma rhodostoma* | *Agkistrodon rhodostoma* | Viperidae | [25] | Type 1/Type 2 | 2 | both | ? | ? | central | III | PA1 |
| *Causus rhombeatus* | *Causus rhombeatus* | Viperidae | [25] | Type 1/Type 2 | 2 | both | ? | ? | central | V | PA1 |
| *Cerastes cerastes* | *Cerastes cornutus* | Viperidae | [25] | Type 1/Type 2 | 2 | both | ? | ? | central | IV | PA1 |
| *Crotalus atrox* | *Crotalus atrox* | Viperidae | [23] | Type 1 | 2 | both | ? | ? | central | I | PA1 |
| *Crotalus durissus* | *Crotalus durissus* | Viperidae | [25] | Type 1/Type 2 | 2 | both | ? | ? | central | I | PA1 |
| *Crotalus durissus* | *Crotalus durissus* | Viperidae | [4] | Type 2 | 2 | both | ? | ? | central |  | PA3 |
| *Crotalus durissus* | *Crotalus durissus* | Viperidae | [27] | ? | 2? | both? | 2? | both? | ? |  | PA1? |
| *Crotalus horridus horridus* | *Crotalus horridus horridus* | Viperidae | [23] | Type 2 | 2 | both | ? | ? | central | I | PA1? |
| *Crotalus scutulatus* | *Crotalus scutulatus* | Viperidae | [25] | Type 1/Type 2 | 2 | both | ? | ? | central | I | PA1 |
| *Crotalus viridis* | *Crotalus viridis* | Viperidae | [5] | Type 1/Type 2 | 2 | both | 2 | both | central |  | PA1 |
| *Crotalus viridis helleri* | *Crotalus viridis helleri* | Viperidae | [23] | Type 1 | 2 | both | ? | ? | central | I | PA1? |
| *Crotalus viridis viridis* | *Crotalus viridis viridis* | Viperidae | [23] | Type 1 | 2 | both | ? | ? | central | I | PA1? |
| *Daboia russelii* | *Vipera russellii* | Viperidae | [25] | Type 1/Type 2 | 2 | both | ? | ? | central | III | PA1 |
| *Echis carinatus* | *Echis carinatis* | Viperidae | [25] | Type 1/Type 2 | 2 | both | ? | ? | central | III | PA1 |
| *Lachesis muta* | *Lachesis muta* | Viperidae | [23] | Type 2 | 2 | both | ? | ? | central | II | PA1 |
| *Lachesis muta* | *Lachesis mutus* | Viperidae | [25] | Type 1/Type 2 | 2 | both | ? | ? | central | II | PA1 |
| *Ovophis monticola* | *Trimeresurus monticola* | Viperidae | [25] | Type 1/Type 2 | 2 | both | ? | ? | central | III | PA1 |
| *Sistrurus catenatus* | *Sistrurus catenatus* | Viperidae | [25] | Type 1/Type 2 | 2 | both | ? | ? | central | III | PA1 |
| *Sistrurus catenatus* | *Sistrurus catenatus* | Viperidae | [23] | Type 1 | 2 | both | ? | ? | central | IIIB | PA1 |
| *Sistrurus catenatus tergeminus* | *Sistrurus catenatus tergeminus* | Viperidae | [23] | Type 1 | 2 | both | ? | ? | central | IIIB | PA1 |
| *Sistrurus miliarus* | *Sistrurus miliarus* | Viperidae | [23] | Type 1 | 2 | both | ? | ? | central | IIIB | PA1 |
| *Sistrurus miliarus barbouri* | *Sistrurus miliarus barbouri* | Viperidae | [23] | Type 1 | 2 | both | ? | ? | central | IIIB | PA1 |
| *Sistrurus ravus* | *Sistrurus ravus* | Viperidae | [23] | Type 2 | 2 | both | ? | ? | central | I | PA1? |
| *Trimeresurus albolabris* | *Trimeresurus albolabris* | Viperidae | [25] | Type 1/Type 2 | 2 | both | ? | ? | central | III | PA1 |
| *Trimeresurus flavoviridis* | *Trimeresurus flavoviridis* | Viperidae | [25] | Type 1/Type 2 | 2 | both | ? | ? | central | III | PA1 |
| *Trimeresurus puniceus* | *Trimeresurus puniceus* | Viperidae | [25] | Type 1/Type 2 | 2 | both | ? | ? | central | III | PA1 |
| *Trimeresurus purpureomaculatus* | *Trimeresurus purpureomaculatus* | Viperidae | [25] | Type 1/Type 2 | 2 | both | ? | ? | central | III | PA1 |
| *Trimeresurus wagleri* | *Trimeresurus wagleri* | Viperidae | [25] | Type 1/Type 2 | 2 | both | ? | ? | central | III | PA1 |
| *Trimeresurus wagleri* | *Trimeresurus wagleri* | Viperidae | [4] | Type 2 | 2 | both | ? | ? | central |  | PA3 |
| *Vipera berus* | *Vipera berus* | Viperidae | [25] | Type 1/Type 2 | 2 | both | ? | ? | central | III | PA1 |
| *Xenopeltis unicolor* | *Xenipeltis unicolor* | Xenopeltidae | [28] | Type 3 | ? | ? | 2 | posterior | ? |  | PV2 |
| *Xenopeltis unicolor* | *Xenopeltis unicolor* | Xenopeltidae | [4] | Type 3 | 2 | posterior | 2 | anterior | anterior |  | PA3 |

**References**

1. Wallach V (1998) The Lungs of Snakes. In: Gans C, Gaunt AS, editors. Biology of the Reptilia Vol.19 (Morphology G). Ithaka, New York: SSAR Press. pp. 93–283.

2. Kashyap H V, Sohoni PR (1973) The heart and arterial system of Acrochordus granulatus. Journal of the University of Bombay 42: 34–52.

3. Brongersma LD (1952) On the tracheal lung and lung in Acrochordus and some other snakes. Archives Néerlandaises de Zoologie 9: 561–562.

4. Brongersma LD (1951) Some remarks on the pulmonary artery in snakes with two lungs. Zoologische Verhandelingen 14: 3–36.

5. Kardong K V (1972) Morphology of the respiratory system and its musculature in different snake genera. Gegenbaurs morphologischen Jahrbuch, Leipzig 117: 285–302.

6. Jensen B, Nyengaard J, Pedersen M, Wang T (2010) Anatomy of the python heart. Anatomical Science International 85: 194–203.

7. Jacquart H (1855) Mémoire sur les organes de la circulation chez le serpent Python. Annales des sciences naturelles 4: 321–364.

8. Starck JM (2009) Functional Morphology and Patterns of Blood Flow in the Heart of Python regius. Journal of Morphology 687: 673–687.

9. Hopkinson JP, Pancoast J (1837) On the Visceral Anatomy of the Python (Cuvier), Described by Daudin as the Boa Reticulata. Transactions of the American Philosophical Society 5: 121– 134.

10. Brongersma LD (1951) Some notes upon the anatomy of Tropidophis and Trachyboa (Serpentes). Zoologische mededelingen 31: 107–124.

11. Atwood WH (1916) The visceral anatomy of the blacksnake (Zamenis constrictor). Washington University Studies 4: 3–38.

12. Brongersma LD (1957) Notes upon the trachea, lungs, and the pulmonary artery in snakes III. Proceedings of the Koninklijke Nederlandse Akademie van Wetenschappen Series C 60: 451–457.

13. Donald JA, O’Shea JE, Lillywhite HB (1990) Neural regulation of the pulmonary vasculature in a semi-arboreal snake, Elaphe obsoleta. Journal of comparative physiology B, Biochemical, systemic, and environmental physiology 159: 677–685.

14. Beddard FE (1906) Contributions to the knowledge of the vascular and respiratory systems in the Ophidia, and to the anatomy of the genera Boa and Corallus. Proceedings of the Zoological Society of London. London. pp. 499–532.

15. O’Donoghue CH (1912) The circulatory system of the common grass snake. Proceedings of the Zoological Society of London. pp. 612–647.

16. Ray HC (1934) On the arterial system of the common Indian rat-snake, Ptyas Mucosus (Linn.). Journal of Morphology 56: 533–575.

17. Ray HC (1936) On the venous system of the common Indian rat-snake Ptyas mucosus (Linn.). Journal of Morphology 59: 517–547. doi:10.1002/jmor.1050590306.

18. Atwood WH (1918) The visceral anatomy of the Garter snake. Transactions of the Wisconsin Academy of Sciences, Arts and Letters 19: 531–552.

19. Kashyap H V (1959) The Reptilian Heart. Proceedings of the National Institute of Sciences of India 26B: 234–254.

20. De Silva PHDH (1953) The arterial system in Ceylon snakes – naja naja naja. Spolia Zeylanica 27: 47–58.

21. Robb J (1960) The internal anatomy of Typhlops Schneider (Reptilia). Australian Journal of Zoology 8: 181–216.

22. Kashyap H V (1950) The structure of the heart of Typhlops (Reptilia: Ophidia). The Journal of the Zoological Society of India 2: 42–48.

23. Van Bourgondien TM, Bothner RC (1969) A Comparative Study of the Arterial Systems of Some New World Crotalinae (Reptilia: Ophidia). American Midland Naturalist 81: 107–147.

24. Bothner RC (1959) The gross anatomy of the heart and neighboring vessels in the northern subspecies of the copperhead, Agkistrodon contortrix mokeson (Daudin). Science Studies 20.

25. Brongersma LD (1949) On the main branches of the pulmonary artery in some viperidae. Bijdragen tot de Dierkunde 28: 57–64.

26. Beddard FE (1906) Contributions to the anatomy of the Ophidia. Proceedings of the Zoological Society of London. London. pp. 12–44.

27. Jensen B, Abe AS, Andrade D V, Nyengaard J, Wang T (2010) The Heart of the South American Rattlesnake, Crotalus durissus. Journal of Morphology 271: 1066–1077.

28. Thompson JC (1913) Contributions to the anatomy of the ophidia. Proceedings of the Zoological Society London 2: 412–425.
